# Gene expression profile of muscle adaptation to high-intensity intermittent exercise training in young men

**DOI:** 10.1038/s41598-018-35115-x

**Published:** 2018-11-14

**Authors:** Eri Miyamoto-Mikami, Katsunori Tsuji, Naoki Horii, Natsuki Hasegawa, Shumpei Fujie, Toshiyuki Homma, Masataka Uchida, Takafumi Hamaoka, Hiroaki Kanehisa, Izumi Tabata, Motoyuki Iemitsu

**Affiliations:** 1 Department of Sports and Life Science, National Institute of Fitness and Sports in Kanoya, Kanoya-city, Kagoshima, Japan; 2 Faculty of Sport and Health Science, Ritsumeikan University, Kusatsu-city, Shiga, Japan; 3 Japan Society for the Promotion of Science, Chiyoda-ku, Tokyo, Japan; 4 Department of Sports Science, Faculty of Sports and Health Science, Daito Bunka University, Higashimatsuyama-city, Saitama, Japan; 5 Department of Sports Medicine for Health Promotion, Tokyo Medical University, Shinjuku-ku, Tokyo, Japan

## Abstract

High-intensity intermittent exercise training (HIIT) has been proposed as an effective approach for improving both, the aerobic and anaerobic exercise capacity. However, the detailed molecular response of the skeletal muscle to HIIT remains unknown. We examined the effects of the HIIT on the global gene expression in the human skeletal muscle. Eleven young healthy men participated in the study and completed a 6-week HIIT program involving exhaustive 6–7 sets of 20-s cycling periods with 10-s rests. In addition to determining the maximal oxygen uptake (${\dot{{\rm{V}}}{\rm{O}}}_{2{\rm{\max }}}$), maximal accumulated oxygen deficit, and thigh muscle cross-sectional area (CSA), muscle biopsy samples were obtained from the vastus lateralis before and after the training to analyse the skeletal muscle transcriptome. The HIIT program significantly increased the ${\dot{{\rm{V}}}{\rm{O}}}_{2{\rm{\max }}}$, maximal accumulated oxygen deficit, and thigh muscle CSA. The expression of 79 genes was significantly elevated (fold-change >1.2), and that of 73 genes was significantly reduced (fold-change <0.8) after HIIT. Gene ontology analysis of the up-regulated genes revealed that the significantly enriched categories were “glucose metabolism”, “extracellular matrix”, “angiogenesis”, and “mitochondrial membrane”. By providing information about a set of genes in the human skeletal muscle that responds to the HIIT, the study provided insight into the mechanism of skeletal muscle adaptation to HIIT.

## Introduction

High-intensity interval/intermittent exercise training (HIIT) has been broadly defined as repeated bouts of short- to moderate-duration exercise completed at high intensity, interspersed with periods of low-intensity exercise or rest^1,2^. There are various types of HIIT, which differ in exercise intensity and in the combinations of exercise and rest periods. Among them, a supramaximal HIIT, consisting of six to seven 20-s exercises at about 170% maximal oxygen uptake (${\dot{{\rm{V}}}{\rm{O}}}_{2{\rm{\max }}}$) intensity interspersed with rest periods of 10 s, stressed both anaerobic and aerobic energy releasing systems almost maximally, although the total duration of this exercise regime was less than 4 min^3^. Furthermore, it has been shown that performing the supramaximal HIIT for 6 weeks significantly elevated the capacity of both aerobic and anaerobic energy releasing systems, and the observed increase in aerobic capacity was comparable to that induced by conventional endurance training, despite a reduction in total exercise volume^4^. These findings support the conclusion that the supramaximal HIIT is a unique training method that not only effectively improves the aerobic energy releasing capacity, but also enhances anaerobic energy releasing capacity.

Multiple studies have examined the molecular mechanisms underlying the exercise-induced adaptation of the skeletal muscle during conventional endurance and resistance training. These studies have identified signalling pathways, and transcription and translation regulators that mediate these adaptations. For example, in endurance training, mitochondrial biogenesis, fatty acid metabolism, and angiogenesis, among others, have been shown to be involved, whereas protein synthesis/degradation and myogenesis, among others, are associated with the adaptation of the muscle in resistance training modules^5^. It has been shown that HIIT induces skeletal muscle adaptations, as observed e.g. elevated levels of the markers of mitochondrial content and oxidative capacity^6,7^, glycolytic capacity^8,9^, intramuscular glycogen and triglyceride stores^7–9^, and capillary density^9–11^. Regarding the molecular mechanisms that underlie the skeletal muscle adaptations to HIIT, previous studies have demonstrated the involvement of the activation of the mitochondrial biogenesis-related signalling pathway^1,12–14^. However, only limited numbers of genes and proteins have been studied in this context, and therefore, the underlying mechanisms of human muscle adaptation to HIIT are not completely understood. A two-dimensional differential gel electrophoresis-based proteomic analysis of skeletal muscles of rats that underwent HIIT demonstrated altered relative abundance of 13 among the 800 proteins detected. However, given that around 20,000 proteins are expressed in the skeletal muscle^15^, such approach does not constitute a comprehensive analysis of the molecular responses to HIIT.

Gene expression profiling is a powerful tool providing new insights into the molecular mechanisms of the muscle adaptation to exercise. In previous studies, gene expression profiles of the skeletal muscle adaptation to both, the endurance and resistance training programmes have been examined. The studies revealed that each programme induces markedly different responses in the muscle, leading to gains in aerobic capacity in the case of endurance training and muscle strength in the case of resistance training^16–18^. Most recently, Robinson *et al*. examined comprehensive gene expression changes induced by HIIT; in that study, HIIT consisted of exercise bouts of lower intensity and longer duration (four periods of 4-min cycling at >90% peak oxygen consumption with 3-min pedalling at no load) compared to the supramaximal HIIT, and was especially targeted for improving the aerobic capacity, mitochondrial respiration, and insulin sensitivity, but not for improving the anaerobic capacity^19^.

Analysis of global gene expression in skeletal muscle before and after the supramaximal HIIT would clarify the detailed molecular mechanisms that underlie the skeletal muscle adaptation to supramaximal HIIT. Specifically, such a study would facilitate the identification of potential genes that contribute to the improvement of anaerobic capacity in HIIT. Therefore, the purpose of the present study was to examine the effects of the supramaximal HIIT on the global gene expression in the human skeletal muscle. Because the skeletal muscle adaptations to exercise training are modulated by several factors, such as sex^20^, age^19^, and training status^2^, the subjects in the current study were only untrained, young, and healthy men. We conducted a microarray analysis of the vastus lateralis (VL) tissue collected before and after a 6-week HIIT in these subjects to identify genes with expression altered by the supramaximal HIIT.

## Results

### Characteristics of the study subjects

**Figure 1:**
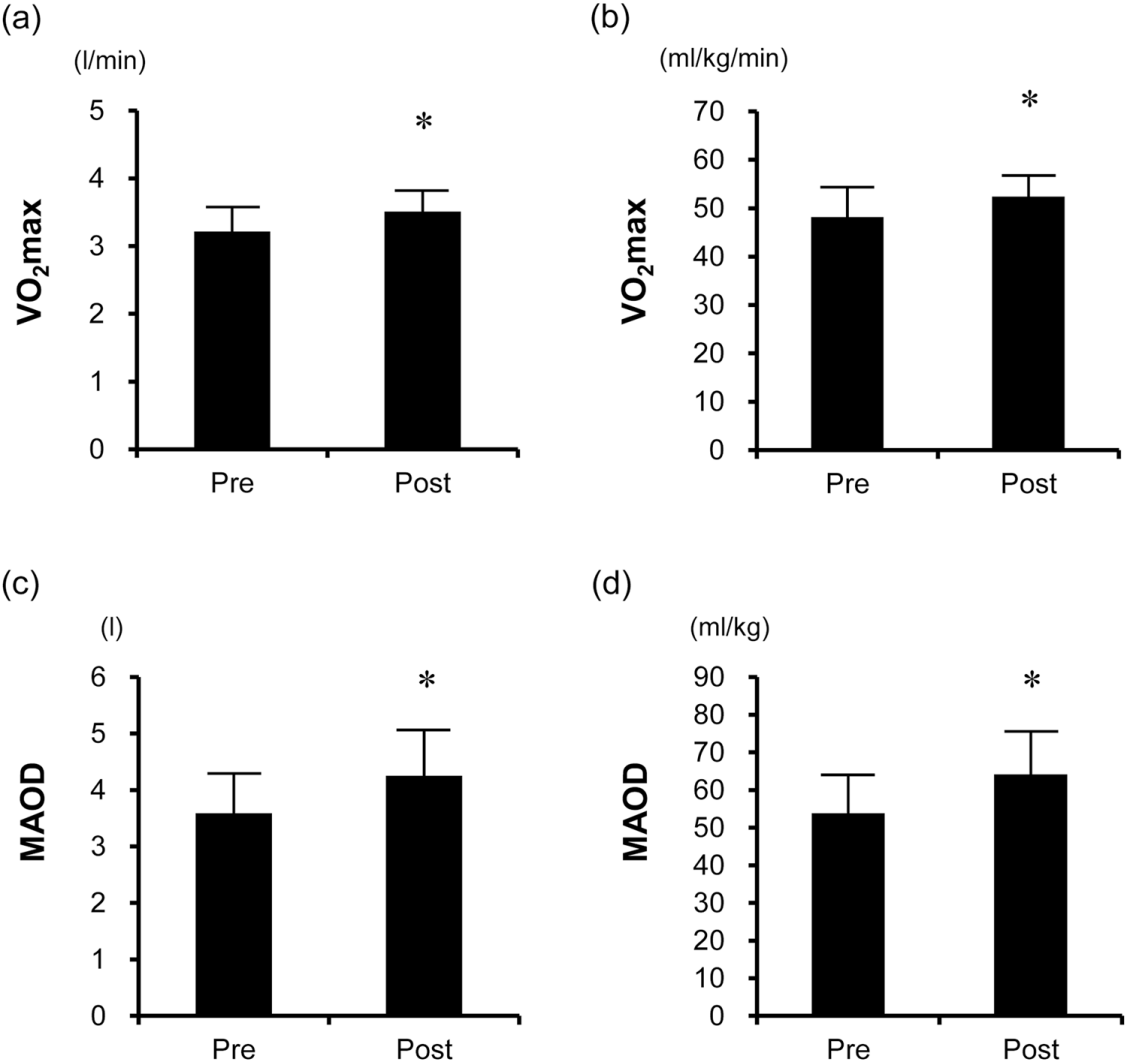
Maximal oxygen uptake [absolute (**a**) and relative (**b**)] and maximal accumulated oxygen deficit [absolute (**c**) and relative (**d**)] before and after HIIT (n = 11). The 6-week HIIT intervention significantly increased ${\dot{{\rm{V}}}{\rm{O}}}_{2{\rm{\max }}}$ and the maximal accumulated oxygen deficit. The bars represent means and SDs. ${\dot{{\rm{V}}}{\rm{O}}}_{2{\rm{\max }}}$: maximal oxygen uptake; MAOD: maximal accumulated oxygen deficit. *P < 0.05 vs. before-HIIT values.

Eleven healthy young men participated in this study. Body weight, body mass index (BMI), whole-body fat percentage (%fat), and whole-body skeletal muscle mass did not change after the 6-week HIIT (Table 1). The cross-sectional areas (CSAs) of the quadriceps femoris and the hamstrings significantly increased after the HIIT (*P* < 0.05), while those of the adductors did not significantly change (*P* = 0.435) (Table 1). The 6-week HIIT intervention significantly increased the maximal oxygen uptake (${\dot{{\rm{V}}}{\rm{O}}}_{2{\rm{\max }}}$) and maximal accumulated oxygen deficit, both the absolute and relative values (*P* < 0.01, Fig. 1). Furthermore, the mean power output elicited during a 40-s maximal sprint test increased after the HIIT (Before training: 573.7 ± 64.1 W vs. After training: 600.6 ± 59.2 W, *P* < 0.01). The nutrient intake was not affected by the HIIT (Table 1).

**Table 1:** Subject characteristics before and after the 6-week HIIT (n = 11).

	Before	After	*P*-value
Age (yr)	23.3 ± 2.8	—	
Height (cm)	173.7 ± 7.2	—	
Body weight (kg)	67.1 ± 7.1	67.7 ± 6.8	0.303
BMI	22.2 ± 1.6	22.4 ± 1.4	0.264
%fat (%)	15.0 ± 1.7	15.6 ± 2.5	0.189
Skeletal muscle mass (kg)	32.4 ± 3.3	32.3 ± 3.2	0.821
Quadriceps femoris CSA (mm^2^)	7059.8 ± 924.8	7358.4 ± 977.7	**0.011**
Hamstrings CSA (mm^2^)	3512.8 ± 588.2	3594.3 ± 599.3	**0.047**
Adductors CSA (mm^2^)	3539.0 ± 793.4	3616.6 ± 830.1	0.435
Total energy intake (kcal/day)	2329.7 ± 719.6	2264.3 ± 766.9	0.446
Protein intake (g/day)	77.7 ± 27.2	77.7 ± 41.1	0.995
Fat intake (g/day)	64.2 ± 30.1	64.7 ± 37.4	0.913
Carbohydrate intake (g/day)	340.6 ± 98.6	318.6 ± 77.4	0.088

Values are presented as mean ± SD. CSA: cross-sectional area.

### Effect of HIIT on the global gene expression in the muscle

Among the 24,838 genes covered by the Human Gene 2.0 ST Array, the expression of 79 genes was significantly up-regulated [fold-change >1.2, false discovery rate (FDR) <0.05, Table 2] and that of 73 was significantly down-regulated (fold-change <0.8, FDR <0.05, Table 3) after the HIIT. Gene ontology (GO) analysis of the functional characteristics of these genes revealed that the genes corresponding to *glucose metabolism*, *extracellular matrix (ECM) organization*, *angiogenesis*, and *mitochondrial membrane* were significantly enriched among the up-regulated genes (Supplemental Table S1). The genes corresponding to *contractile fibre*, *regulation of synaptic transmission, mitochondrial matrix*, and *cytoskeletal protein binding* were significantly enriched among the down-regulated genes (Supplemental Table S2). Pathway analysis revealed that the up- and down-regulated genes were significantly associated with 23 (Supplemental Table S3) and 12 (Supplemental Table S4) pathways, respectively. As shown in Supplemental Table S3, *glucose metabolism*, *gluconeogenesis*, *focal adhesion*, *arginine and proline metabolism*, and *PI3K-Akt signalling pathway* were the top five significantly enriched pathways associated with the up-regulated genes.

**Table 2:** Seventy-nine genes that were significantly up-regulated after 6-week HIIT.

Gene symbol	Gene description	Expression value		Fold change	*P-*value
		Before	After		
*ADAMTS15*	ADAM metallopeptidase with thrombospondin type 1 motif, 15	28.5	34.9	1.23	0.00146
*ATP5G3*	ATP synthase, H + transporting, mitochondrial Fo complex, subunit C3 (subunit 9)	278.5	343.3	1.23	0.00013
*BCAS2*	breast carcinoma amplified sequence 2	283.3	390.6	1.38	0.00050
*C2orf57*	chromosome 2 open reading frame 57	30.8	37.5	1.22	0.00236
*C3orf35*	chromosome 3 open reading frame 35	68.7	83.3	1.21	0.00030
*CA14*	carbonic anhydrase XIV	65.6	130.3	1.99	0.00022
*CARNS1*	carnosine synthase 1	122.5	156.9	1.28	5.6E-05
*CD79A*	CD79a molecule, immunoglobulin-associated alpha	60.8	98.3	1.62	3.4E-05
*CKMT2*	creatine kinase, mitochondrial 2 (sarcomeric)	933.8	1194.8	1.28	0.00044
*COL4A1*	collagen, type IV, alpha 1	72.9	106.6	1.46	0.00022
*COL4A2*	collagen, type IV, alpha 2	75.2	96.2	1.28	0.00059
*DENND2C*	DENN/MADD domain containing 2C	459.3	582.8	1.27	0.00142
*DNM1P35*	DNM1 pseudogene 35	62.4	76.3	1.22	0.00027
*DSTNP2*	destrin (actin depolymerizing factor) pseudogene 2	31.4	43.8	1.40	0.00147
*DUX4L9*	double homeobox 4 like 9	76.9	93.2	1.21	0.00187
*FARP1*	FERM, RhoGEF (ARHGEF) and pleckstrin domain protein 1 (chondrocyte-derived)	61.2	81.8	1.34	0.00047
*FBP2*	fructose-1,6-bisphosphatase 2	1292.5	1726.0	1.34	1E-05
*FGF6*	fibroblast growth factor 6	43.9	59.5	1.35	2.5E-06
*FLJ31813*	FAM21B pseudogene	41.6	52.3	1.26	0.00152
*G0S2*	G0/G1switch 2	448.4	644.1	1.44	0.00106
*GADL1*	glutamate decarboxylase-like 1	143.3	202.0	1.41	5.7E-05
*GOT1*	glutamic-oxaloacetic transaminase 1, soluble (aspartate aminotransferase 1)	1324.3	1612.1	1.22	0.00105
*HIST3H2BB*	histone cluster 3, H2bb	57.8	69.7	1.21	0.00125
*IRX3*	iroquoishomeobox 3	96.6	117.3	1.21	0.00013
*KDR*	kinase insert domain receptor (a type III receptor tyrosine kinase)	69.3	90.6	1.31	0.00022
*LAMB1*	laminin, beta 1	56.4	82.2	1.46	9.1E-05
*LOC100131174*	uncharacterized LOC100131174	103.1	251.6	2.44	1.5E-05
*LOC100506114*	uncharacterized LOC100506114	662.2	876.1	1.32	0.00063
*LOC283693*	actin, alpha 2, smooth muscle, aorta pseudogene	32.5	40.5	1.25	0.00053
*LXN*	latexin	39.6	48.8	1.23	0.00215
*MDH1*	malate dehydrogenase 1, NAD (soluble)	555.5	671.4	1.21	0.00037
*MIR106B*	microRNA 106b	50.2	60.8	1.21	0.00186
*MIR346*	microRNA 346	116.6	143.7	1.23	0.00017
*MIR378I*	microRNA 378i	53.7	67.2	1.25	0.00067
*MIR412*	microRNA 412	63.6	79.4	1.25	0.00161
*MIR4442*	microRNA 4442	379.6	480.7	1.27	0.00020
*MXD3*	MAX dimerization protein 3	31.4	39.3	1.25	0.00149
*MYLK4*	myosin light chain kinase family, member 4	177.4	270.3	1.52	0.00019
*MYO19*	myosin XIX	65.5	80.5	1.23	0.00169
*NETO2*	neuropilin (NRP) and tolloid (TLL)-like 2	46.8	63.8	1.36	0.00067
*NINL*	ninein-like	35.3	42.5	1.20	0.00250
*NMRK2*	nicotinamide riboside kinase 2	211.6	924.4	4.37	2.9E-07
*NRP1*	neuropilin 1	232.1	285.1	1.23	0.00113
*OSBPL7*	oxysterol binding protein-like 7	66.5	85.6	1.29	2.9E-06
*PDE4C*	phosphodiesterase 4C, cAMP-specific	44.1	55.8	1.26	0.00093
*PGK1*	phosphoglycerate kinase 1	213.2	256.9	1.21	4.7E-05
*PKM*	pyruvate kinase, muscle	1368.4	1792.2	1.31	3E-05
*PPARGC1A*	peroxisome proliferator-activated receptor gamma, coactivator 1 alpha	215.5	263.2	1.22	0.00190
*PPP1R3C*	protein phosphatase 1, regulatory subunit 3C	1914.0	2377.1	1.24	0.00066
*PRND*	prion protein 2 (dublet)	31.7	42.7	1.35	0.00121
*PROC*	protein C (inactivator of coagulation factors Va and VIIIa)	40.7	49.0	1.20	0.00164
*PXDN*	peroxidasin homolog (Drosophila)	44.0	54.8	1.25	0.00159
*RPL13P5*	ribosomal protein L13 pseudogene 5	68.3	95.5	1.40	6.1E-05
*SCN4B*	sodium channel, voltage-gated, type IV, beta subunit	63.9	78.2	1.22	0.00253
*SF3A3*	splicing factor 3a, subunit 3, 60 kDa	244.4	459.9	1.88	1.9E-05
*SGK1*	serum/glucocorticoid regulated kinase 1	53.9	72.7	1.35	0.00085
*SIPA1L2*	signal-induced proliferation-associated 1 like 2	69.3	88.1	1.27	0.00068
*SLC18B1*	solute carrier family 18, subfamily B, member 1	60.2	74.4	1.24	0.00037
*SLC25A15*	solute carrier family 25 (mitochondrial carrier; ornithine transporter) member 15	32.7	39.4	1.21	0.00041
*SLC25A30*	solute carrier family 25, member 30	383.6	527.5	1.37	0.00176
*SNORA80B*	small nucleolar RNA, H/ACA box 80B	61.5	77.3	1.26	0.00110
*SNORD115-1*	small nucleolar RNA, C/D box 115-1	170.2	245.5	1.44	0.00077
*SNORD115-11*	small nucleolar RNA, C/D box 115-11	172.0	275.4	1.60	0.00120
*SNORD115-12*	small nucleolar RNA, C/D box 115-12	172.0	275.4	1.60	0.00120
*SNORD115-20*	small nucleolar RNA, C/D box 115-20	95.6	155.5	1.63	0.00018
*SNORD115-22*	small nucleolar RNA, C/D box 115-22	160.9	246.9	1.53	0.00224
*SNORD115-39*	small nucleolar RNA, C/D box 115-39	181.2	274.1	1.51	0.00017
*SNORD115-42*	small nucleolar RNA, C/D box 115-42	167.4	266.3	1.59	0.00137
*SNRPN*	small nuclear ribonucleoprotein polypeptide N	30.7	39.9	1.30	0.00097
*SNTB1*	syntrophin, beta 1 (dystrophin-associated protein A1, 59 kDa, basic component 1)	103.2	126.2	1.22	0.00190
*SREBF1*	sterol regulatory element binding transcription factor 1	38.8	48.2	1.24	0.00092
*SYT11*	synaptotagmin XI	65.2	80.6	1.24	0.00083
*TMEM70*	transmembrane protein 70	78.1	104.4	1.34	0.00026
*TMIE*	transmembrane inner ear	49.7	60.4	1.22	0.00124
*TPSAB1*	tryptase alpha/beta 1	51.9	63.5	1.22	0.00072
*TRBV5-6*	T cell receptor beta variable 5-6	68.6	88.2	1.29	0.00014
*ZCCHC9*	zinc finger, CCHC domain containing 9	65.5	102.3	1.56	8.1E-07
*ZNF700*	zinc finger protein 700	28.8	34.9	1.21	0.00151
*ZNF91*	zinc finger protein 91	126.0	167.5	1.33	0.00041

**Table 3:** Seventy-three genes that were significantly down-regulated after 6-week HIIT.

Gene symbol	Gene description	Expression value		Fold change	*P*-value
		Before	After		
*AASS*	aminoadipate-semialdehyde synthase	167.2	128.6	0.77	0.00022
*ADHFE1*	alcohol dehydrogenase, iron containing, 1	382.8	305.8	0.80	0.00018
*ALDH2*	aldehyde dehydrogenase 2 family (mitochondrial)	249.8	178.3	0.71	2.27E-06
*ALDH6A1*	aldehyde dehydrogenase 6 family, member A1	130.0	103.2	0.79	0.00027
*ASB15*	ankyrin repeat and SOCS box containing 15	542.1	378.8	0.70	9.14E-07
*B3GALT1*	UDP-Gal:betaGlcNAc beta 1,3-galactosyltransferase, polypeptide 1	120.3	80.9	0.67	9.89E-05
*C3orf26*	chromosome 3 open reading frame 26	70.7	54.3	0.77	0.00012
*C8orf22*	chromosome 8 open reading frame 22	2864.8	2091.7	0.73	0.00074
*CADM2*	cell adhesion molecule 2	54.7	42.8	0.78	3.48E-04
*CCDC141*	coiled-coil domain containing 141	45.9	36.3	0.79	0.00184
*CD38*	CD38 molecule	287.3	224.0	0.78	0.00139
*DHCR24*	24-dehydrocholesterol reductase	112.4	83.6	0.74	7.05E-06
*DMGDH*	dimethylglycine dehydrogenase	56.2	43.8	0.78	0.00041
*EFR3A*	EFR3 homolog A (S. cerevisiae)	458.1	364.8	0.80	1.13E-04
*EGFR*	epidermal growth factor receptor	128.2	101.5	0.79	7.09E-05
*EPB41*	erythrocyte membrane protein band 4.1 (elliptocytosis 1, RH-linked)	183.4	144.8	0.79	0.00058
*EYA1*	eyes absent homolog 1 (Drosophila)	143.1	111.0	0.78	1.62E-05
*FAM126A*	family with sequence similarity 126, member A	162.7	114.5	0.70	1.01E-03
*FBXO48*	F-box protein 48	80.1	60.8	0.76	0.00267
*FLJ43663*	uncharacterized LOC378805	149.3	102.7	0.69	2.56E-04
*GATSL1*	GATS protein-like 1	423.2	336.8	0.80	0.00011
*GPT2*	glutamic pyruvate transaminase (alanine aminotransferase) 2	143.9	106.6	0.74	0.00017
*HIST2H2AC*	histone cluster 2, H2ac	1263.4	957.1	0.76	0.00175
*HN1*	hematological and neurological expressed 1	61.1	48.3	0.79	4.14E-05
*HSPB1*	heat shock 27 kDa protein 1	1910.4	1494.8	0.78	8.53E-05
*HSPB3*	heat shock 27 kDa protein 3	271.0	211.9	0.78	0.00019
*IFIT1*	interferon-induced protein with tetratricopeptide repeats 1	56.0	41.0	0.73	0.00012
*IL17D*	interleukin 17D	119.3	94.1	0.79	0.00030
*IL32*	interleukin 32	82.0	55.0	0.67	5.80E-05
*KIF1B*	kinesin family member 1B	437.7	343.4	0.78	0.00028
*LGI1*	leucine-rich, glioma inactivated 1	77.2	57.5	0.75	0.00036
*LGR5*	leucine-rich repeat containing G protein-coupled receptor 5	360.0	214.3	0.60	0.00083
*LMOD1*	leiomodin 1 (smooth muscle)	76.3	58.9	0.77	4.23E-05
*LOC100128560*	uncharacterized LOC100128560	56.2	37.5	0.67	0.00207
*LOC100188947*	uncharacterized LOC100188947	67.1	45.2	0.67	0.00103
*LOC100505769*	uncharacterized LOC100505769	100.8	78.9	0.78	0.00242
*LOC100506338*	uncharacterized LOC100506338	75.9	60.0	0.79	1.10E-04
*LOC646903*	uncharacterized LOC646903	172.2	123.4	0.72	0.00016
*MFAP4*	microfibrillar-associated protein 4	209.4	152.8	0.73	0.00167
*MIR4681*	microRNA 4681	65.3	51.6	0.79	4.59E-05
*MKNK2*	MAP kinase interacting serine/threonine kinase 2	287.6	221.2	0.77	0.00017
*MSTN*	myostatin	139.8	73.6	0.53	6.92E-06
*MT1X*	metallothionein 1×	1524.8	1169.2	0.77	0.00108
*MYH1*	myosin, heavy chain 1, skeletal muscle, adult	4283.1	2788.7	0.65	0.00233
*MYLK2*	myosin light chain kinase 2	701.4	534.4	0.76	0.00148
*MYOM3*	myomesin family, member 3	282.3	190.2	0.67	5.58E-05
*NEK10*	NIMA (never in mitosis gene a)- related kinase 10	145.5	108.4	0.74	9.22E-05
*NRAP*	nebulin-related anchoring protein	4720.4	3508.3	0.74	1.91E-06
*NT5C2*	5′-nucleotidase, cytosolic II	413.8	311.6	0.75	0.00077
*NUP160*	nucleoporin 160 kDa	84.1	66.5	0.79	5.87E-04
*OXCT1*	3-oxoacid CoA transferase 1	115.0	79.9	0.70	0.00101
*PAIP2B*	poly(A) binding protein interacting protein 2B	517.1	382.1	0.74	0.00039
*PDLIM3*	PDZ and LIM domain 3	2003.3	1553.8	0.78	0.00012
*PFN2*	profilin 2	243.7	189.8	0.78	9.41E-05
*POLR2J*	polymerase (RNA) II (DNA directed) polypeptide J, 13.3 kDa	109.9	78.1	0.71	0.00026
*PRKAG3*	protein kinase, AMP-activated, gamma 3 non-catalytic subunit	194.4	145.4	0.75	0.00035
*PYGO1*	pygopus homolog 1 (Drosophila)	129.5	100.3	0.77	0.00051
*SF3A1*	splicing factor 3a, subunit 1, 120 kDa	129.0	103.0	0.80	1.08E-04
*SH3RF2*	SH3 domain containing ring finger 2	104.6	75.9	0.73	6.20E-05
*SLC1A3*	solute carrier family 1 (glial high affinity glutamate transporter), member 3	61.7	49.3	0.80	0.00142
*SLC25A33*	solute carrier family 25 (pyrimidine nucleotide carrier), member 33	147.8	113.2	0.77	0.00201
*SLITRK4*	SLIT and NTRK-like family, member 4	70.5	52.8	0.75	0.00243
*SMTNL1*	smoothelin-like 1	318.2	184.2	0.58	4.52E-04
*TMEM47*	transmembrane protein 47	544.1	433.1	0.80	0.00039
*TP63*	tumor protein p63	136.6	90.8	0.67	8.80E-04
*TSC22D1*	TSC22 domain family, member 1	185.1	142.2	0.77	0.00034
*UNC13B*	unc-13 homolog B (C. elegans)	194.8	155.9	0.80	0.00036
*UNC13C*	unc-13 homolog C (C. elegans)	61.8	48.2	0.78	0.00132
*WBP11*	WW domain binding protein 11	94.3	74.5	0.79	0.00121
*WDR5B*	WD repeat domain 5B	50.8	40.6	0.80	0.00092
*YIPF7*	Yip1 domain family, member 7	295.9	235.9	0.80	0.00055
*ZFP36*	zinc finger protein 36, C3H type, homolog (mouse)	118.9	94.1	0.79	0.00023
*ZNF844*	zinc finger protein 844	65.9	51.8	0.79	0.00065

### Validation of a subset of genes by reverse transcription quantitative polymerase chain reaction (RT-qPCR)

**Figure 2:**
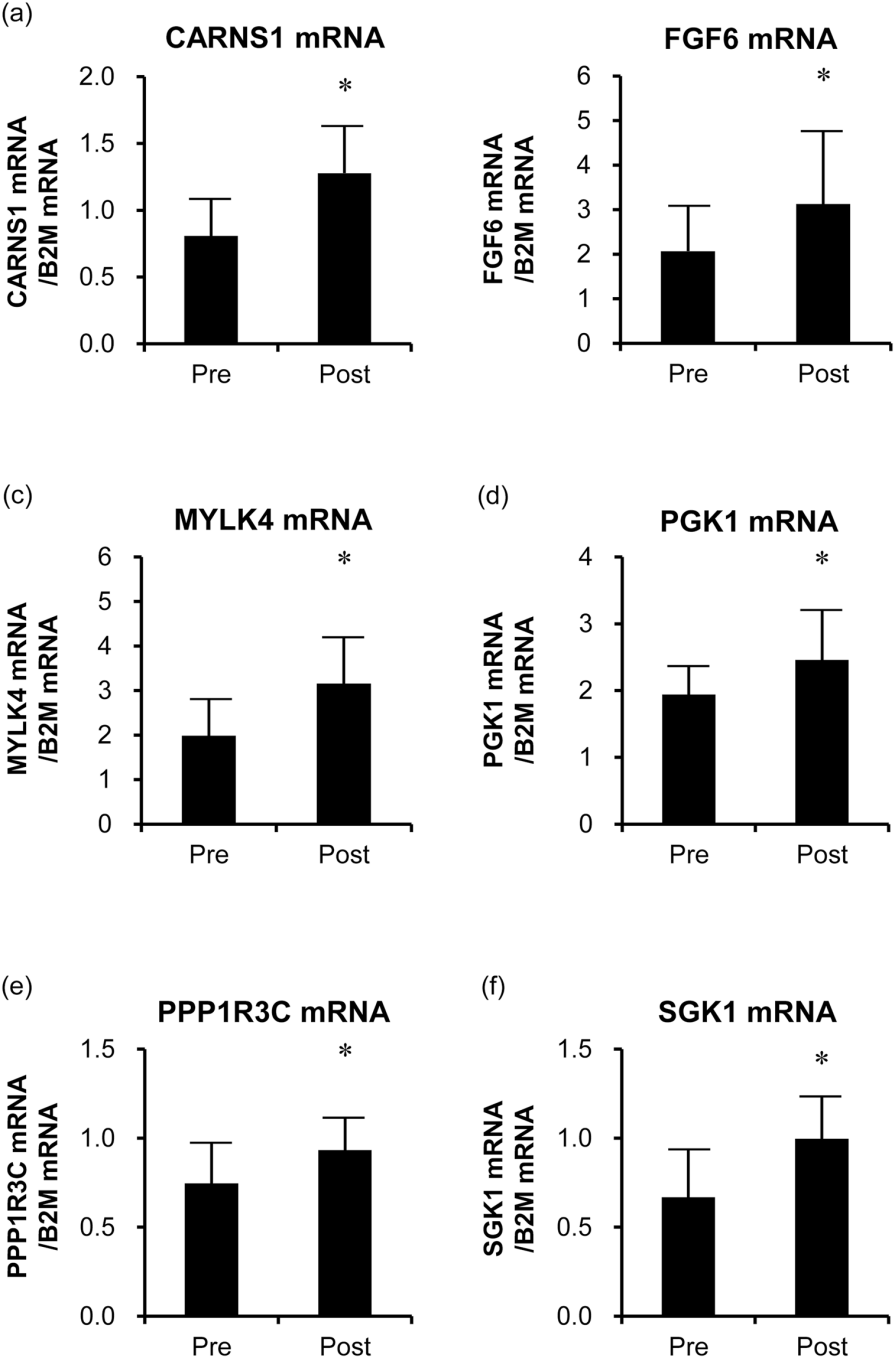
The expression of *CARNS1* (**a**), *FGF6* (**b**), *MYLK4* (**c)**, *PGK1* (**d**), *PPP1R3C* (**e**), and *SGK1* (**f**) genes in the skeletal muscle before and after HIIT (n = 11). The mRNA levels of the six genes were determined by quantitative PCR and significantly increased after the HIIT. The bars represent means and SDs. The *B2M* mRNA was used as an internal control. *P < 0.05 vs. before-HIIT values.

To confirm the validity of the microarray data, we examined the associations of the mRNA expression values with those obtained by RT-qPCR. Among the 79 up-regulated genes, we focused on seven genes that were related to both the significantly enriched GO categories and the significantly enriched pathways (Supplemental Tables S1 and S3). Among them, we first selected gene for the peroxisome proliferator-activated receptor gamma, coactivator 1 alpha (*PPARGC1A*), whose product has been reported to be associated with the HIIT^21,22^. As indicated by the microarray results, mRNA levels of *PPARGC1A* significantly increased after the HIIT (fold-change = 1.49, *P* = 0.002). To identify novel exercise-related genes, we selected six genes whose induction by exercise or training had not been reported, namely, ones encoding: carnosine synthase 1 (*CARNS1*); fibroblast growth factor 6 (*FGF6*); myosin light chain kinase family, member 4 (*MYLK4*); phosphoglycerate kinase 1 (*PGK1*); protein phosphatase 1, regulatory subunit 3 C (*PPP1R3C*); and serum/glucocorticoid regulated kinase 1 (*SGK1*). RT-qPCR analyses confirmed that the expression of these genes was significantly increased after HIIT (Fig. 2). For these genes, the expression values obtained by microarray analysis were positively associated with those obtained by RT-qPCR (*CARNS1*, *r* = 0.64, *FGF6*, *r* = 0.73, *MYLK4*, *r* = 0.73, *PGK1*, *r* = 0.50, *PPP1R3C*, *r* = 0.79, and *SGK1*, *r* = 0.84, *P* *<* 0.05).

### HIIT-associated proteins and enzyme activities

**Figure 3:**
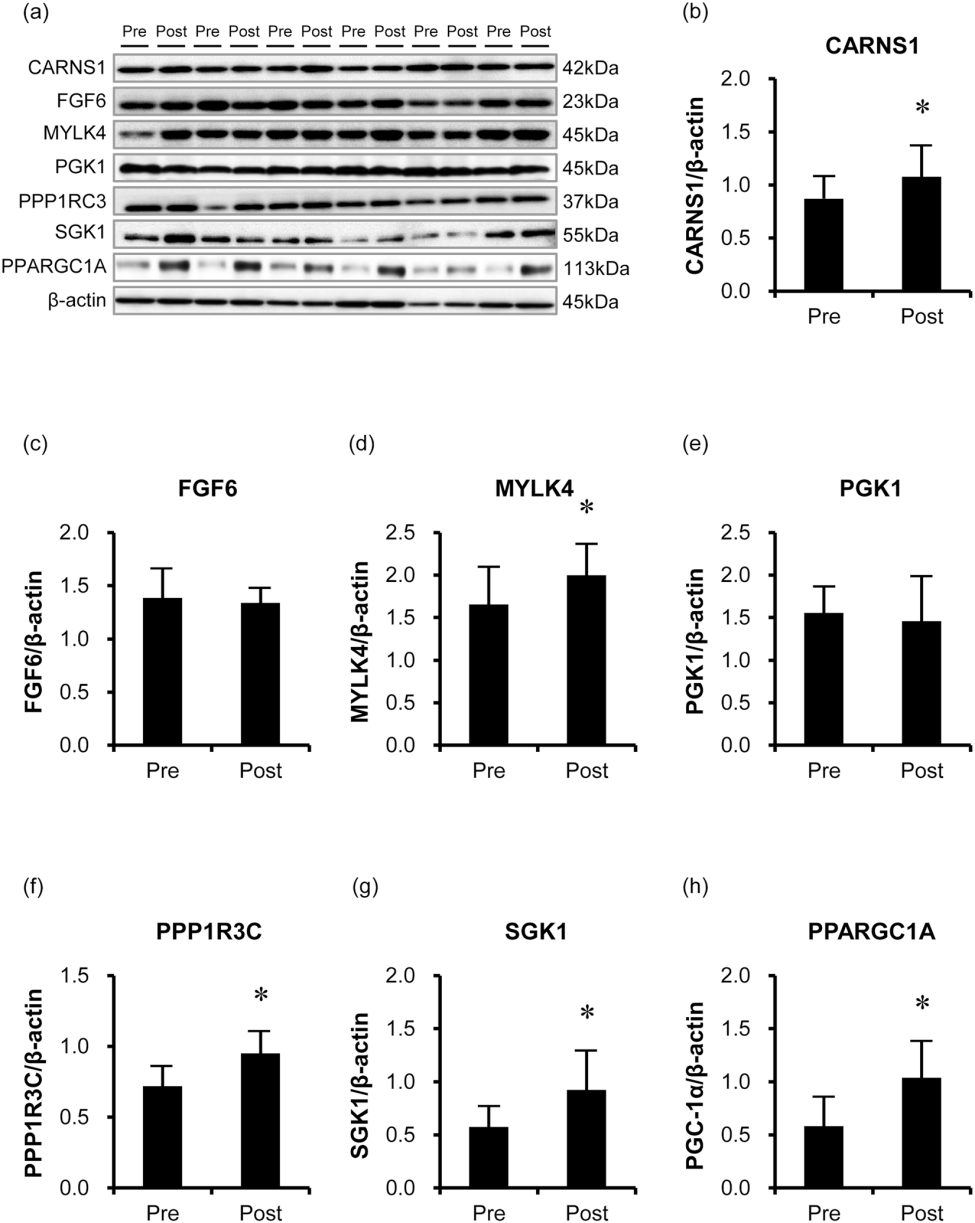
Representative immunoblotting image (**a**) and protein expression levels of CARNS1 (**b**), FGF6 (**c**), MYLK4 (**d**), PGK1 (**e**), PPP1R3C (**f**), SGK1 (**g**), and PPARGC1A (**h**) in the skeletal muscle before and after HIIT (n = 11). CARNS1, MYLK4, PPP1R3C, SGK1, and PPARGC1A levels significantly increased after the HIIT, while those of FGF6 and PGK1 did not change. The bars represent means and SDs. A representative western blot is shown. β-actin protein was used as an internal control. *P < 0.05 vs. before-HIIT values. Full image of the gels are presented in Supplemental Figure S1.

**Figure 4:**
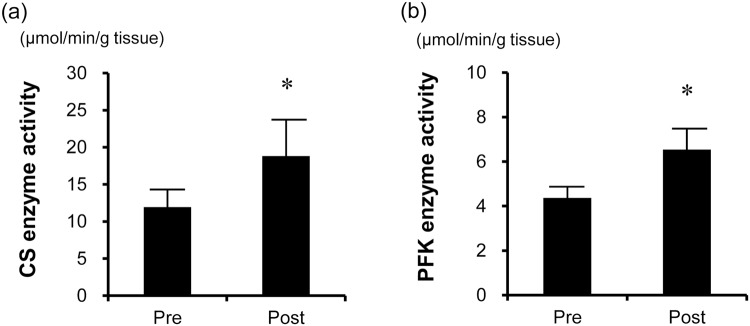
Enzyme activities of CS (**a**) and PFK (**b**) (n = 11). The activities of CS and PFK significantly increased after the HIIT. The bars represent means and SDs. *P < 0.05 vs. before-HIIT values.

To confirm the translational responses of the identified HIIT-responsive genes to the HIIT, the levels and enzymatic activities of specific proteins were evaluated. Protein levels of CARNS1, MYLK4, PPP1R3C, SGK1 and PPARGC1A were significantly increased after the HIIT (*P* < 0.05), while those of FGF6 (*P* = 0.478) and PGK1 (*P* = 0.498) did not change (Fig. 3). Furthermore, citrate synthase (CS) and phosphofructokinase (PFK) activities, which have previously been reported to be elevated by HIIT, were significantly increased after the HIIT (*P* < 0.01, Fig. 4).

## Discussion

The 6-week supramaximal HIIT significantly improved both the anaerobic and aerobic capacities, as reported previously^4^. The microarray data obtained in the current study constitute the transcriptome signature of the post-HIIT adaptations of the human skeletal muscle. After the HIIT, specific subset of genes were significantly up- and down-regulated. The GO and pathway analyses of the up-regulated genes revealed novel exercise-related genes (*CARNS1*, *FGF6*, *MYLK4*, *PGK1*, *PPP1R3C*, and *SGK1*). The validity of microarray data for these genes was subsequently confirmed by RT-qPCR. Further, the levels of proteins encoded by four genes (*CARNS1*, *MYLK4*, *PPP1R3C*, and *SGK1*) were significantly increased by HIIT, which suggested that the genes whose expression appeared to have been altered by the HIIT may be responsible, at least in part, for the physiological adaptations of the skeletal muscle to the supramaximal HIIT.

### Comparison of gene expression profiles for HIIT and other types of training

PPARGC1A protein level and CS activity in the skeletal muscle were significantly elevated after HIIT. This was consistent with previous findings that HIIT activates mitochondrial biogenesis-related signalling pathways linked to PPARGC1A^12,13^ and that HIIT increases mitochondrial enzyme levels and activities^6,13,23^ in the human skeletal muscle. Collectively, these observations indicated that HIIT induces mitochondrial biogenesis in the skeletal muscle. Since that process (mitochondrial biogenesis) responds similarly to endurance training, it is reasonable to assume that the two training methods elicit common or overlapping gene expression changes in the skeletal muscle. Indeed, among the 79 HIIT-induced genes, 13 (*CA14*, *COL4A1*, *COL4A2*, *FARP1*, *GOT1*, *KDR*, *LAMB1*, *LXN*, *NRP1*, *PXDN*, *SCN4B*, *SIPA1L2*, and *TPSAB1*) showed altered expression with endurance training^16^, although at different significance levels. On the other hand, Robinson *et al*. reported that 30% of HIIT-induced genes are shared with resistance training-induced genes^19^. In the current study, among the 79 HIIT-induced genes, we identified seven genes (*COL4A1*, *COL4A2*, *KDR*, *LAMB1*, *LXN*, *NETO2*, and *PRND*) whose expression was increased by resistance training^18^. However, when comparing the results of different studies, we should keep in mind the differences in intervention periods, training frequency, study locations, and so on, between the studies.

In terms of GO, *glucose metabolism*, *ECM organization*, *angiogenesis*, and *mitochondrial membrane* are examples of significantly enriched categories among the HIIT-induced genes identified in the current study. Previous studies showed that endurance and resistance training enhance the expression of ECM-related genes^16,18^. ECM is known to be involved in signal transduction^24,25^ and cushioning of the myofibres from mechanical strain^26^. Based on the results of the current and previous studies, it can be stated that ECM remodelling occurs during exercise training, regardless of the exercise mode (i.e., endurance, resistance, and high-intensity interval/intermittent).

### Candidate genes involved in the muscle adaptation to HIIT

An increase in maximal accumulated oxygen deficit is an adaptation specific to supramaximal HIIT^4^. Anaerobic capacity assessed on the basis of maximal accumulated oxygen deficit can be defined as the maximal amount of ATP formed by breakdown of phosphocreatine and glycolysis in a working skeletal muscle during exercise. Thus, the amount of creatine phosphate and glycolytic enzyme activity in the skeletal muscle influence the anaerobic capacity. However, little is known about the molecular mechanisms responsible for such improvement of the anaerobic capacity. In the current study, the activity of PFK, the rate-limiting enzyme of glycolysis, was increased after HIIT. This agrees with previous studies in humans^8,27^. Therefore, it is likely that facilitation of glycolysis in the skeletal muscle contributes to the increase in anaerobic capacity associated with HIIT.

Muscle glycogen is an important fuel for the working muscle, especially during high-intensity exercise. Indeed, it has been shown that HIIT significantly reduces glycogen concentration in the human skeletal muscle^6,28^. After high-intensity exercise, sensitization of the insulin-stimulated glucose transport response and activation of glycogen synthase (GS) augment glycogen re-synthesis^29^. It is possible that two HIIT-induced genes, *SGK1* and *PPP1R3C*, are involved in these adaptations.

SGK1 plays an important role in insulin-dependent glucose uptake in the skeletal muscle^30^. In comparison with wild-type mouse, *SGK1* knockout mouse reportedly exhibits a significant reduction in muscle glucose uptake following intraperitoneal glucose injection^30^. In the current study, the SGK1 levels increased after HIIT. It has been previously reported that HIIT increases insulin sensitivity of the skeletal muscle, assessed as the rate of glucose disappearance during a hyperinsulinemic-euglycemic clamp^19^. The increased glucose uptake activity of the skeletal muscle has been considered to reflect enhanced GLUT4 protein production after HIIT^13,31^. However, considering previous findings in knockout mice^30^ and the observed HIIT-induced increase in SGK1 protein level, SGK1 might also contribute to the increase in insulin-stimulated glucose uptake in the skeletal muscle after HIIT.

Further, mRNA and protein levels of PPP1R3C were significantly increased in the skeletal muscle after HIIT. PPP1R3C is a protein phosphatase-1 glycogen-targeting subunit (PP1-GTS) that regulates glycogen metabolism^32^. Although the relationship between this protein and exercise metabolism remains unclear, PPP1R3A is required for the activation of GS that occurs in the skeletal muscle after exercise^33^. The basal glycogen levels in the skeletal muscle in *PPP1R3A* knockout mouse are significantly reduced and the maximal exercise capacity is impaired, although muscle contraction-induced activation of glucose transport remains unaffected^33^. Overexpression of *PPP1R3C* more strongly promotes GS protein production and activation in the skeletal muscle cells than the overexpression of *PPP1R3A*^34^. Considering all of the above, it is reasonable to propose that PPP1R3C contributes to the regulation of glycogen synthesis in the skeletal muscle after HIIT.

Microarray analysis presented in the current study that *CARNS1* expression was significantly increased after HIIT. CARNS1 catalyses the formation of carnosine from l-histidine and β-alanine in the skeletal muscle. Carnosine is mainly present in the skeletal muscle tissues of mammals^35^ and plays various roles, such as proton buffering, protecting against reactive oxygen uptake, and regulating calcium handling^36^. Previous studies showed that the carnosine content of the skeletal muscle is associated with high-intensity exercise performance^37,38^, and that sprinters have a higher muscle carnosine content than endurance runners and untrained individuals^39,40^. In the present study, the 40-s maximal sprint performance increased after the HIIT. Therefore, we speculate that the increase in CARNS1 protein levels in the skeletal muscle upon HIIT observed in the current study may be linked to an increase in muscle carnosine content.

Myosin light chain kinase phosphorylates the regulatory light chain (RLC) of sarcomeric myosin. Evidence from a variety of muscle models suggests that phosphorylation of myosin RLC alters the myosin motor structure. Further, alterations in the myosin structure increase the rate of force generation by myosin cross-bridges to increase Ca^2+^-sensitivity of the contractile apparatus^41^. *MYLK2* encodes a skeletal muscle isoform of myosin light chain kinase. In the present study, however, *MYLK2* gene expression in the skeletal muscle was reduced after the HIIT (fold change = 0.76, *P* = 0.0005), while *MYLK4* gene expression and protein levels were significantly increased after the HIIT. Although the function and role of MYLK4 in the skeletal muscle have not yet been elucidated, it is known that *MYLK4* is expressed in the human heart, and that *MYLK4* expression and protein levels are significantly decreased in patients with heart failure^42^. Further investigation is warranted to elucidate the role of MYLK4 increase in muscle contraction and exercise performance.

Microarray and RT-qPCR analyses presented in the current study confirmed the HIIT-induced increases in *FGF6* and *PGK1* gene expression. However, the protein levels of FGF6 and PGK1 did not change after HIIT, suggesting post-transcriptional regulation of these genes^43^.

In addition to the specific six genes whose expression was investigated in detail in the current study, genes whose functions are not known and genes that had not been linked to exercise to date were identified as HIIT-response genes. To elucidate the role of these genes, we examined the correlation between changes in gene expression levels and changes in physiological parameters. Although it is a limitation that there are several steps between gene expression and physiological functions, we found that changes in the expression of several genes were strongly correlated with changes in the physiological parameters (Supplemental Tables S5 and S6). Further research on their functions and relationship with exercise will contribute to clarifying the underlying mechanisms of skeletal muscle adaptation to HIIT.

There are several limitations to the current study. First, it did not include a control group as such. It is therefore possible that certain genes might have been induced by factors other than HIIT, such as the muscle biopsy, and altered physical activity and/or diet patterns. Regarding muscle biopsy as a possible confounding factor, it was previously shown to not cause transcriptional changes in the muscle^44^. The subjects were reminded to not alter their physical activity level and dietary habits for the duration of the study. Indeed, their nutrient intake did not change, as assessed by a brief self-administered diet history questionnaire before and after HIIT. Although we are convinced that the changes in gene expression reported in the current study reflected the effects of HIIT, future studies should include an appropriate control group. Another limitation of this study is that the effects of HIIT on global gene expression were only examined in men. One cannot exclude the possibility that HIIT-associated gene expression alterations in women would differ from the ones evidenced herein. Future studies should examine the effects of the supramaximal HIIT on skeletal muscles in women. Finally, in the present study, global gene expression was compared only before and after the 6-week HIIT intervention. Perry *et al*. (2010) reported that the timing and magnitude of mRNA responses to HIIT varied depending on the type of gene^45^. Therefore, it is necessary to examine the time course of global gene expression response in human skeletal muscles during an HIIT intervention.

In conclusion, elucidating the molecular bases of training adaptation will ultimately provide clues for future and novel training methodologies. In the current study, we identified a set of genes in the human skeletal muscle that responds to the supramaximal HIIT. The HIIT-induced genes included those that possibly contribute to the increase in the anaerobic and aerobic capacities (Supplemental Figure S2). These findings will help to elucidate the molecular mechanisms underlying physiological adaptation of the muscle to HIIT. Future investigations, e.g. functional analyses, are warranted to provide insight into their roles in the human skeletal muscle adaptation to exercise and physical performance.

## Materials and Methods

### Subjects and ethical approval

Eleven young, healthy men voluntarily participated in this study. All participants were given an oral and written briefing of the study, and each of the subjects provided written informed consent before the study. The study was approved by the Ethics Committees of the Ritsumeikan University and was conducted in accordance with the Declaration of Helsinki. The subjects were sedentary or moderately active and did not participate in vigorous sport activities. All subjects did not have chronic diseases and did not take any medications; did not drink large quantities of alcohol (average alcohol intake: 38.0 g/week), and were reminded throughout the study not to alter their physical activity levels or dietary habits for the duration of the study. A brief self-administered diet history questionnaire^46^ was used to assess their dietary intake.

### Study design

All participants underwent a 6-week HIIT program. Body composition, ${\dot{{\rm{V}}}{\rm{O}}}_{2{\rm{\max }}}$, and the maximal accumulated oxygen deficit were determined and a 40-s maximal sprint test was conducted before and after the 6-week HIIT. Muscle biopsy samples were collected from the VL before and after the 6-week HIIT. In the current study, all exercise tests, as well as HIIT, were conducted on a mechanically braked cycle ergometer (828E, Monark, Vansbro, Sweden) at a pedalling frequency of 90 rpm. Exercise tests before and after the HIIT were carried out on two to three separate days.

### Body composition

Body weight and height were measured to the nearest 0.1 cm and 0.1 kg, respectively, and BMI (kg/m^2^) was calculated using these two variables. The %fat and skeletal muscle mass were assessed using an InBody 770 body composition analyser (Biospace, Tokyo, Japan). The thigh muscle CSAs were assessed using magnetic resonance imaging (Signa HDxt, 1.5 T; GE Healthcare UK, Little Chalfont, UK), as previously described, with some modifications^47,48^. A series of axial slice images (10-mm-thick) was obtained from the superior border of the patella to the anterior superior iliac spine. The magnetic resonance image at 50% of the thigh length was used for the CSA analysis. CSAs of the quadriceps femoris, hamstrings, and adductors were determined using an image analysis software (Slice-o-matic v 4.3 for Windows, Tomovision, Magog, QC, Canada). For each analysed muscle, the muscle boundary was traced manually three times, and the average of three measurements was adopted as the representative CSA value. All muscle CSA analyses were performed by one examiner. The average coefficient of variation for the CSA analyses was 0.64 ± 0.39%.

### Muscle biopsy

Pre-training muscle biopsies were conducted before any exercise testing, and the subjects were instructed to avoid exercise 24 h prior to these biopsies. Post-training muscle biopsies were conducted 48–72 h after the last training session, and the subjects were instructed to avoid exercise from the end of the last training session until the muscle biopsies. The subjects arrived in the laboratory in the morning after an overnight fast. The muscle biopsies were performed as previously described^48^. Briefly, two muscle biopsy samples (10–20 mg each) were obtained from the lateral portion of the VL at 2-cm depth from the fascia using a 14 G biopsy needle (Bard Max-Core; C. R. Bard, Tempe, AZ, USA) under sterile conditions with local anaesthesia (1% lidocaine). One sample was used for microarray and RT-qPCR analyses; the other sample was used for immunoblotting and enzyme activity analyses. To avoid sampling from a previously damaged area, the direction of the needle stab was slightly altered for the second biopsy. The muscle samples were quickly rinsed with ice-cold saline, cleaned to remove any visible non-muscle material, and frozen immediately in liquid nitrogen. The samples were stored at –80 °C until use.

### ${\dot{{\rm{V}}}{\rm{O}}}_{2{\rm{\max }}}$

To determine the linear relationship between exercise intensity and steady-state oxygen uptake, each subject conducted six to nine 10-min exercises at constant power (between 35% and 90% ${\dot{{\rm{V}}}{\rm{O}}}_{2{\rm{\max }}}$). All exercises were conducted at a pedalling frequency of 90 rpm. During the exercise, the heart rate was monitored continuously. The expired air was collected during the last 1–2 min of each exercise using the Douglas-bag method. The oxygen and carbon dioxide fractions in the expired air were measured using a mass spectrometer (ARCO-2000; ARCO System, Chiba, Japan). The gas volume was measured using a dry gas meter (DC-2; Shinagawa Seisakusho, Tokyo, Japan). A linear relationship between exercise intensity and steady-state oxygen uptake was determined for each subject^3^. The linearity of the relationship for individual subjects were verified using linear regression and correlation analyses (r = 0.997 ± 0.004, n = 11).

After a 2-min warm-up exercise at approximately 70–80% ${\dot{{\rm{V}}}{\rm{O}}}_{2{\rm{\max }}}$, a supramaximal intensity exercise at 90 rpm that exhausted the subjects within 2–4 min was conducted. Oxygen uptake was measured for the last two or three 30-s intervals of the supramaximal exercise using the Douglas-bag method as mentioned above. This exercise was conducted several times at different intensities. After confirming levelling-off of the oxygen uptake with increasing intensity, the highest VO_2_ among the trials was designated as the ${\dot{{\rm{V}}}{\rm{O}}}_{2{\rm{\max }}}$^49^.

### Maximal accumulated oxygen deficit

The maximal accumulated oxygen deficit was determined as an index of anaerobic capacity during a 2–3-min exhaustive bicycle exercise, in accordance with the method described by Medbø *et al*.^50^. Prior to the test trial, the subjects were asked to perform a 10-min warm-up at about 50% of ${\dot{{\rm{V}}}{\rm{O}}}_{2{\rm{\max }}}$. In the test trial, the subjects exercised at the pre-set intensity of 90 rpm to exhaustion (defined as the state in which they were unable to maintain the pedalling rate above 85 rpm despite strong verbal encouragement). The exercise intensity was chosen individually for each subject, to cause exhaustion within 2–3 min. The expired air was collected over the entire exercise duration and the accumulated oxygen uptake was measured using the Douglas-bag method. The oxygen demand of the exhausting exercise was estimated by extrapolating from the linear relationship between exercise intensity and the steady-state oxygen uptake to the power used during the experiment. The accumulated oxygen demand was calculated as the product of the estimated oxygen demand and the duration of the exercise. The accumulated oxygen deficit was calculated as the difference between the accumulated oxygen demand and the accumulated oxygen uptake.

### 40-s maximal sprint test

All subjects completed a warm-up exercise comprising a 30-s submaximal sprint (at load equal to 3% of body weight) and a 5-s maximal sprint (at load equal to 5% of body weight) on an electromagnetically braked cycle ergometer (Power Max VIII; Konami Corp., Tokyo, Japan). After the warm-up, subjects performed a 40-s all-out effort on the electromagnetically braked cycle ergometer against a resistance equivalent to 7.5% of their body weight. Subjects were instructed to start pedalling as fast as possible and verbally encouraged to keep pedalling as fast as possible throughout the 40-s maximal sprint test. The power output was recorded during the sprint test.

### HIIT intervention

All subjects completed short-lasting, exhaustive 6-week HIIT (100% compliance)^4^, including 24 training sessions (4 d/week). Each session consisted of six to seven sets of 20-s cycling on a leg ergometer (828E, Monark) at an intensity of about 170% of ${\dot{{\rm{V}}}{\rm{O}}}_{2{\rm{\max }}}$ at 90 rpm, with a 10-s rest between each bout. A linear extrapolation to higher power was performed to determine 170% ${\dot{{\rm{V}}}{\rm{O}}}_{2{\rm{\max }}}$ and the corresponding bicycling exercise intensity using the established relationship between the power and steady state oxygen uptake described above. An O_2_ demand of 170% ${\dot{{\rm{V}}}{\rm{O}}}_{2{\rm{\max }}}$ was considered to be 1.70 times ${\dot{{\rm{V}}}{\rm{O}}}_{2{\rm{\max }}}$ (L/min), and the corresponding biking power was determined from the linear relationship (see Fig. 1 in Tabata *et al*.^3^). The subjects were encouraged to complete six to seven sets of the exercise. The exercise was terminated when the pedalling frequency dropped below 85 rpm for about 5 s, despite strong verbal encouragement. When the subjects completed more than seven exercise sets, the exercise intensity was increased by 11 W.

### Microarray analysis

Total RNA was isolated from the muscle biopsy samples using the miRNeasy^®^ mini kit (Qiagen, Hilden, Germany), and RNA quality was checked using an Agilent 2100 Bioanalyzer (Agilent Technologies, Santa Clara, CA, USA). Fragmented and labelled cDNA samples were prepared from 500 ng of RNA using GeneChip^®^ WT PLUS reagent kit (Affymetrix, Santa Clara, CA, USA) according to the manufacturer’s instructions. The labelled cDNA was hybridized to the Human Gene 2.0 ST Array (Affymetrix). The arrays were washed and stained using the GeneChip^®^ Fluidics Station 450 (Affymetrix), and were scanned in the GeneChip^®^ Scanner 3000 7 G (Affymetrix). Digitalization of the image data and normalization were conducted using Affymetrix^®^ Expression Console software. The microarray data are available at the NCBI Gene Expression Omnibus under the accession number GSE109657.

### RT-qPCR

To validate the microarray data, an aliquot of the total RNA was used in RT-qPCR reactions to evaluate the expression of seven genes: *CARNS1*, *FGF6*, *MYLK4*, *PGK1*, *PPARGC1A*, *PPP1R3C*, and *SGK1*. Six genes (all but *PPARGC1A*) were selected to identify novel exercise-related genes according to the following criteria: 1) genes related to both significantly (*P* < 0.05) enriched GO categories and pathways and 2) genes that had not been reported as induced by exercise or training. *PPARGC1A* was selected to confirm the association with HIIT noted in previous studies^21,22^. Total RNA was reverse-transcribed using Omniscript RT kit (Qiagen) in a 20-μL reaction mixture containing the oligo(dT)_12–18_ primer (Invitrogen, Carlsbad, CA, USA). The mRNA levels were analysed according to a method described previously^48^, using TaqMan gene expression assays (assay IDs: *CARNS1* [Hs00325412_m1], *FGF6* [Hs00173934_m1], *MYLK4* [Hs01584150_m1], *PGK1* [Hs00943178_g1], *PPARGC1A* [Hs01016719_m1], *PPP1R3C* [Hs01921501_s1], and *SGK1* [Hs00985033_g1]) and the StepOne^TM^ Real-Time PCR system (Life Technologies Japan, Tokyo, Japan). Beta-2 microglobulin (*B2M*: Hs00187842-m1) was used as an internal expression control^7,51^. PCR was performed in a 12.5-μL reaction mixture containing TaqMan^®^ universal master mix II with UNG, TaqMan^®^ gene expression assay mix, and cDNA (from 10 ng of total RNA). All reactions were performed in duplicate. Standard curves were generated for each gene by using five different concentrations of total RNA in duplicate. The expression levels of the seven genes were normalized to the expression levels of *B2M* mRNA.

### Immunoblotting

The muscle samples were homogenized in a buffer containing 20 mM Tris-HCl (pH7.8), 300 mM NaCl, 2 mM EDTA, 2 mM dithothreitol, 2% Nonidet P-40, 0.2% sodium dodecyl sulphate, 0.2% sodium deoxycholate, 0.5 mM phenylmethane sulfonyl fluoride, 60 mg/mL aprotinin, and 1 mg/ml leupeptin. The homogenates were slowly rotated for 30 min at 4 °C. They were then centrifuged at 12,000 × g for 15 min at 4 °C. The supernatants were used for western blotting analysis, as previously described^47^. Briefly, the muscle proteins (15 µg) were separated on 10% sodium dodecyl sulphate-polyacrylamide gels, and transferred to polyvinylidene difluoride membranes (Millipore, Billerica, MA, USA). The membranes were treated with a blocking buffer [5% skimmed milk in phosphate-buffered saline with 0.1% Tween-20 (PBS-T)] for 60 min at room temperature. They were then incubated for 12 h at 4 °C in blocking buffer containing the desired antibodies (diluted 1:1000 in the blocking buffer) against CARNS1 (ab167240; Abcam, Tokyo, Japan), FGF6 (ab103479; Abcam), MYLK4 (ab179395; Abcam), PGK1 (ab199438; Abcam), PPARGC1A (ST1202; Millipore), PPP1R3C (ab103300; Abcam), and SGK1 (ab59337; Abcam). β-actin (#4967; Cell Signaling Technology, Beverly, MA) was used as a loading control. The membranes were washed three times with PBS-T and then incubated for 1 h at room temperature with horseradish peroxidase-conjugated secondary antibody and anti-rabbit (GE Healthcare Biosciences, Piscataway, NJ, USA), or anti-mouse (GE Healthcare Biosciences) antibodies diluted 1:3000 in the blocking buffer. For SGK1 detection, the secondary antibody was diluted 1:5000 in another blocking buffer (3% skimmed milk in PBS-T). The membranes were then washed again three times with PBS-T. Finally, the protein levels were detected using an Enhanced Chemiluminescence Plus system with Image Quant LAS4000 (GE Healthcare Biosciences) and quantified by densitometry using Image Quant TL 7.0 software (GE Healthcare Biosciences).

### Enzyme activities

The PFK enzyme activity was measured in the cytosolic fraction of the muscle tissue that had been prepared for immunoblotting. Each sample was incubated in 1 M potassium fluoride in an incubation mixture containing 25 mM β-glycerophosphate, 100 µM DTT, 12 mM glycylglycine, 0.2 mM fructose 6-phosphate, 0.5 mM ATP, and 0.1 mM β-NADH for 5 min at 30 °C. The reaction was monitored at 340 nm for 5 min using a spectrophotometer^52^.

To determine the CS enzyme activity, mitochondrial-enriched fractions were prepared as previously described^53^. The muscle samples were homogenized in 10 volumes of 250 mM sucrose, 1 mM Tris-HCl (pH 7.4), and 130 mM NaCl on ice using a Teflon homogenizer. The homogenate was centrifuged at 9000 × *g* for 20 min at 0 °C; and the pellet was re-suspended in the homogenate buffer and centrifuged again at 600 × *g* for 10 min at 0 °C. The supernatant was centrifuged at 8000 × *g* for 15 min at 0 °C, and the pellet was re-suspended in 250 mM sucrose. Then, 50 µL of each sample was incubated for 2 min at 30 °C in a 900-µL incubation mixture containing 100 mM Tris-HCl (pH 8.0), 1 mM 5,5′-dithiobis (2-nitrobenzoic acid), and 10 mM acetyl-CoA. The reaction was initiated by adding 50 µL of 10 mM oxaloacetate and recorded in a spectrophotometer at 412 nm for 3 min^53^.

### Statistics and microarray data analysis

Values were expressed as means ± standard deviations (SDs). For each parameter, the difference between the values before and after the intervention period was tested by paired Student’s *t*-tests. Pearson’s product-moment correlation coefficient was used to determine the relationship. The statistical significance threshold was set at *P* *<* 0.05 for all comparisons, except for the microarray data (see below). These analyses were performed using SPSS v 22 (SPSS Inc., Chicago, IL, USA).

Microarray data were analysed using the Microarray Data Analysis Tool v 3.2 (Filgen, Aichi, Japan). To exclude data with low reliability, probe sets with values below the average expression value of 3575 negative control probes were excluded. Each expression value was divided by the median expression value of all samples for each probe and log_2_-transformed. The difference between the expression values before and after the training intervention was tested by paired Student’s *t*-tests. The *P* value from each comparison was adjusted for multiple testing using the FDR^54^. Probe sets with a fold-change >1.2 or <0.8 and FDR <0.05 were considered significant and were used in further investigations. GO and pathway analyses of the significantly up- and down-regulated probe sets were conducted with the Microarray Data Analysis Tool v 3.2, which uses the NCBI BioSystems database (http://www.ncbi.nlm.nih.gov/sites/entrez?db=biosystems). For GO analysis, genes expressed in human skeletal muscles, which were selected by determining the genes that had an expression value above the average expression value of the negative control probes, were used as the background gene set. GO categories and pathways with a Z score >0 and *P* < 0.05 were considered significant.

## Electronic supplementary material

Supplemental data
