# Supplementary material for: Gene expression profile of muscle adaptation to high-intensity intermittent exercise training in young men
Source: Sci Rep. 2018 Nov 14;8:16811. doi: 10.1038/s41598-018-35115-x (PMC6235852; doi:10.1038/s41598-018-35115-x)
Supplement: Supplementary file 1 — Supplemental data [file 41598_2018_35115_MOESM1_ESM.pdf]

Gene expression profile of muscle adaptation to high-intensity intermittent exercise training in young men

Eri Miyamoto-Mikami, Katsunori Tsuji, Naoki Horii, Natsuki Hasegawa, Shumpei Fujie, Toshiyuki Homma, Masataka Uchida, Takafumi Hamaoka, Hiroaki Kanehisa, Izumi Tabata, Motoyuki Iemitsu

Supplemental Table S1. GO categories significantly enriched among the HIIT-induced genes

GO term	Total genes*1	Changed genes*2	Z score*3	P-value*4	Gene
gluconeogenesis	41	5	10.394	3.53E-06	FBP2, GOT1, MDH1, PGK1, PPARGC1A
hexose biosynthetic process	43	5	10.127	4.37E-06	FBP2, GOT1, MDH1, PGK1, PPARGC1A
monosaccharide biosynthetic process	48	5	9.533	7.19E-06	FBP2, GOT1, MDH1, PGK1, PPARGC1A
glucose metabolic process	158	7	6.834	2.00E-05	FBP2, GOT1, MDH1, PGK1, PKM, PPARGC1A, PPP1R3C
carbohydrate biosynthetic process	108	6	7.276	2.46E-05	FBP2, GOT1, MDH1, PGK1, PPARGC1A, PPP1R3C
hexose metabolic process	177	7	6.353	4.04E-05	FBP2, GOT1, MDH1, PGK1, PKM, PPARGC1A, PPP1R3C
monosaccharide metabolic process	190	7	6.064	6.25E-05	FBP2, GOT1, MDH1, PGK1, PKM, PPARGC1A, PPP1R3C
extracellular matrix structural constituent	55	4	6.887	0.000252	COL4A1, COL4A2, LAMB1, PXDN
extracellular matrix	253	6	4.336	0.00143	ADAMTS15, COL4A1, COL4A2, LAMB1, PXDN, TPSAB1
basement membrane	56	3	5.265	0.00281	COL4A1, COL4A2, LAMB1
single-organism carbohydrate metabolic process	384	7	3.549	0.00373	FBP2, GOT1, MDH1, PGK1, PKM, PPARGC1A, PPP1R3C
proteinaceous extracellular matrix	214	5	3.914	0.00388	ADAMTS15, COL4A1, COL4A2, LAMB1, PXDN
extracellular region part	573	8	3.156	0.00608	ADAMTS15, COL4A1, COL4A2, FGF6, LAMB1, PROC, PXDN, TPSAB1
positive regulation of epithelial cell proliferation	73	3	4.264	0.00703	KDR, LAMB1, NRP1
angiogenesis	233	5	3.45	0.00746	COL4A1, COL4A2, FGF6, KDR, NRP1
growth factor binding	78	3	4.033	0.00883	COL4A1, KDR, NRP1
kinase activity	576	8	2.838	0.01077	CKMT2, KDR, MYLK4, NMRK2, NRP1, PGK1, PKM, SGK1
mitochondrial inner membrane	282	5	3.128	0.01187	ATP5G3, CKMT2, SLC25A15, SLC25A30, TMEM70
blood vessel morphogenesis	272	5	3.022	0.0138	COL4A1, COL4A2, FGF6, KDR, NRP1
response to external stimulus	743	9	2.619	0.01386	COL4A1, COL4A2, KDR, LAMB1, LXN, NRP1, PPARGC1A, PROC, SREBF1
carbohydrate metabolic process	494	7	2.771	0.01391	FBP2, GOT1, MDH1, PGK1, PKM, PPARGC1A, PPP1R3C
mitochondrial membrane	414	6	2.839	0.01479	ATP5G3, CKMT2, MYO19, SLC25A15, SLC25A30, TMEM70
organelle inner membrane	305	5	2.916	0.01611	ATP5G3, CKMT2, SLC25A15, SLC25A30, TMEM70
single-organism carbohydrate catabolic process	105	3	3.33	0.01819	PGK1, PKM, PPP1R3C
mitochondrial envelope	434	6	2.706	0.01822	ATP5G3, CKMT2, MYO19, SLC25A15, SLC25A30, TMEM70
carbohydrate catabolic process	107	3	3.284	0.01909	PGK1, PKM, PPP1R3C
regulation of cellular component movement	308	5	2.692	0.02223	KDR, LAMB1, NRP1, SCN4B, SGK1
extracellular matrix part	124	3	3.113	0.02293	COL4A1, COL4A2, LAMB1
lyase activity	116	3	3.05	0.02466	CA14, GADL1, GOT1

blood vessel development	317	5	2.617	0.02477	COL4A1, COL4A2, FGF6, KDR, NRP1
small molecule metabolic process	1676	15	2.133	0.02512	ATP5G3, CARNS1, CKMT2, FBP2, G0S2, GADL1, GOT1, MDH1, NMRK2, PDE4C, PGK1, PKM, PPARGC1A, SLC25A15, SREBF1
transferase activity, transferring phosphorus-containing groups	677	8	2.335	0.02584	CKMT2, KDR, MYLK4, NMRK2, NRP1, PGK1, PKM, SGK1
vasculature development	332	5	2.498	0.0294	COL4A1, COL4A2, FGF6, KDR, NRP1
regulation of epithelial cell proliferation	127	3	2.886	0.02945	KDR, LAMB1, NRP1
axon guidance	225	4	2.623	0.03023	COL4A1, COL4A2, LAMB1, NRP1
extracellular region	1039	10	2.233	0.03058	ADAMTS15, COL4A1, COL4A2, FGF6, KDR, LAMB1, NRP1, PROC, PXDN, TPSAB1
extracellular structure organization	131	3	2.817	0.03182	COL4A2, PXDN, TPSAB1
extracellular matrix organization	131	3	2.817	0.03182	COL4A2, PXDN, TPSAB1
axon	142	3	2.804	0.03224	GOT1, IRX3, NRP1
carboxylic acid metabolic process	599	7	2.207	0.03514	CARNS1, CKMT2, GADL1, GOT1, MDH1, PPARGC1A, SLC25A15
generation of precursor metabolites and energy	349	5	2.37	0.03529	MDH1, PGK1, PKM, PPARGC1A, PPP1R3C
chemotaxis	353	5	2.341	0.03677	COL4A1, COL4A2, KDR, LAMB1, NRP1
taxis	353	5	2.341	0.03677	COL4A1, COL4A2, KDR, LAMB1, NRP1
single-organism metabolic process	2051	17	1.944	0.03709	ATP5G3, CARNS1, CKMT2, FBP2, G0S2, GADL1, GOT1, MDH1, NMRK2, PDE4C, PGK1, PKM, PPARGC1A, PPP1R3C, PXDN, SLC25A15, SREBF1
monovalent inorganic cation transport	140	3	2.67	0.03751	ATP5G3, SCN4B, SGK1
alpha-amino acid metabolic process	141	3	2.654	0.03817	CARNS1, CKMT2, GOT1,
organelle envelope	664	7	2.126	0.04011	ATP5G3, CKMT2, MYO19, SLC25A15, SLC25A30, SREBF1, TMEM70
envelope	666	7	2.118	0.04066	ATP5G3, CKMT2, MYO19, SLC25A15, SLC25A30, SREBF1, TMEM70
lipid binding	480	6	2.172	0.04153	ATP5G3, FARP1, LAMB1, OSBPL7, SNTB1, SYT11
organonitrogen compound biosynthetic process	367	5	2.243	0.04227	ATP5G3, CARNS1, GOT1, NMRK2, SLC25A15
epithelial cell proliferation	150	3	2.52	0.04439	KDR, LAMB1, NRP1
oxoacid metabolic process	634	7	2.044	0.04548	CARNS1, CKMT2, GADL1, GOT1, MDH1, PPARGC1A, SLC25A15
organic acid metabolic process	638	7	2.027	0.04677	CARNS1, CKMT2, GADL1, GOT1, MDH1, PPARGC1A, SLC25A15
positive regulation of cell migration	155	3	2.45	0.04806	KDR, LAMB1, NRP1
positive regulation of cell motility	156	3	2.436	0.04881	KDR, LAMB1, NRP1

*1: Number of genes that are linked to each GO term and GO terms located at the hierarchy under the GO among all of the genes expressed in human skeletal muscle.

*2: Number of genes that are linked to each GO term and GO terms located at the hierarchy under the GO among the 79 significantly upregulated genes after the HIIT intervention.

*3: A value indicating by how much the number of changed genes deviates from the expected value based on the ratio of changed genes to total genes expressed in human skeletal muscle.

*4: The significance probability against the null hypothesis that there is no difference between the ratios of genes with specific GO terms to those without specific GO terms between the changed gene set

and the total genes expressed in human skeletal muscle.

Supplemental Table S2. GO categories significantly enriched among the genes downregulated by HIIT

GO term	Total genes*1	Changed genes*2	Z score*3	P-value*4	Gene
myofibril	126	7	7.698	6.39E-06	HSPB1, MYH1, MYLK2, MYOM3, NRAP, PDLIM3, SMTNL1
contractile fiber	133	7	7.448	8.97E-06	HSPB1, MYH1, MYLK2, MYOM3, NRAP, PDLIM3, SMTNL1
sarcomere	106	6	7.209	2.78E-05	HSPB1, MYH1, MYLK2, MYOM3, PDLIM3, SMTNL1
contractile fiber part	120	6	6.681	5.41E-05	HSPB1, MYH1, MYLK2, MYOM3, PDLIM3, SMTNL1
regulation of synaptic transmission	97	5	6.196	2.08E-04	CD38, LGI1, MYLK2, SLC1A3, UNC13B
synaptic transmission	309	8	4.909	0.000262	ALDH2, CD38, KIF1B, LGI1, MYLK2, SLC1A3, UNC13B, UNC13C
regulation of transmission of nerve impulse	105	5	5.897	0.000296	CD38, LGI1, MYLK2, SLC1A3, UNC13B
mitochondrial matrix	240	7	5.037	0.000328	AASS, ADHFE1, ALDH2, ALDH6A1, DMGDH, GPT2, OXCT1
A band	23	3	8.198	0.000347	MYH1, MYOM3, SMTNL1
regulation of neurological system process	110	5	5.727	0.000363	CD38, LGI1, MYLK2, SLC1A3, UNC13B
single-organism catabolic process	175	6	5.208	0.000395	AASS, ALDH2, ALDH6A1, DMGDH, GPT2, OXCT1
small molecule catabolic process	175	6	5.208	0.000395	AASS, ALDH2, ALDH6A1, DMGDH, GPT2, OXCT1
regulation of system process	253	7	4.827	0.000446	CD38, LGI1, MSTN, MYLK2, SLC1A3, SMTNL1, UNC13B
cell-cell signaling	533	10	4.203	0.000536	ALDH2, CD38, KIF1B, LGI1, MYLK2, OXCT1, SLC1A3, TP63, UNC13B, UNC13C
transmission of nerve impulse	355	8	4.399	0.000648	ALDH2, CD38, KIF1B, LGI1, MYLK2, SLC1A3, UNC13B, UNC13C
multicellular organismal signaling	360	8	4.349	0.000709	ALDH2, CD38, KIF1B, LGI1, MYLK2, SLC1A3, UNC13B, UNC13C
system process	890	13	3.741	0.000762	ALDH2, CD38, EPB41, EYA1, KIF1B, LGI1, LMOD1, MSTN, MYLK2, SLC1A3, SMTNL1, UNC13B, UNC13C
cytoskeletal protein binding	461	9	4.136	0.000799	EGFR, EPB41, KIF1B, LMOD1, MYH1, MYLK2, NRAP, PDLIM3, PFN2
alpha-amino acid metabolic process	141	5	4.866	0.00108	AASS, ALDH6A1, DMGDH, GPT2, SLC1A3
cellular amino acid catabolic process	82	4	5.355	0.00114	AASS, ALDH6A1, DMGDH, GPT2
regulation of synaptic plasticity	41	3	5.916	0.00166	CD38, MYLK2, UNC13B
synapse	252	6	3.999	0.00247	CADM2, LGI1, MYLK2, SLC1A3, UNC13B, UNC13C
calmodulin binding	106	4	4.541	0.00282	EPB41, MYH1, MYLK2, SMTNL1
actin binding	267	6	3.805	0.00328	EGFR, EPB41, LMOD1, MYH1, NRAP, PFN2
dicarboxylic acid metabolic process	54	3	5.024	0.0035	ADHFE1, GPT2, SLC1A3
neurological system process	578	9	3.331	0.00371	ALDH2, CD38, EYA1, KIF1B, LGI1, MYLK2, SLC1A3, UNC13B, UNC13C
alpha-amino acid catabolic process	61	3	4.661	0.00485	AASS, DMGDH, GPT2
positive regulation of multicellular	290	6	3.548	0.00488	CD38, HSPB1, LGI1, MYLK2, SLC1A3, SMTNL1

organismal process					
mitochondrial part	619	9	3.114	0.00578	AASS, ADHFE1, ALDH2, ALDH6A1, DMGDH, GPT2, OXCT1, SLC1A3, SLC25A33
regulation of neurotransmitter levels	67	3	4.394	0.00624	ALDH2, SLC1A3, UNC13B
I band	68	3	4.366	0.00643	HSPB1, PDLIM3, SMTNL1
carboxylic acid catabolic process	140	4	3.737	0.00735	AASS, ALDH6A1, DMGDH, GPT2
organic acid catabolic process	140	4	3.737	0.00735	AASS, ALDH6A1, DMGDH, GPT2
cytokine activity	79	3	3.95	0.00962	IL17D, IL32, MSTN
cell junction	452	7	2.937	0.01086	CADM2, LGI1, MYH1, NRAP, TMEM47, UNC13B, UNC13C
signaling	3004	25	2.176	0.01542	ALDH2, ASB15, CD38, DHCR24, EGFR, EYA1, FAM126A, HSPB1, IFIT1, KIF1B, LGI1, LGR5, MFAP4, MKNK2, MSTN, MYLK2, NUP160, OXCT1, PRKAG3, PYGO1, SLC1A3, TP63, UNC13B, UNC13C, ZFP36
single organism signaling	3004	25	2.176	0.01542	ALDH2, ASB15, CD38, DHCR24, EGFR, EYA1, FAM126A, HSPB1, IFIT1, KIF1B, LGI1, LGR5, MFAP4, MKNK2, MSTN, MYLK2, NUP160, OXCT1, PRKAG3, PYGO1, SLC1A3, TP63, UNC13B, UNC13C, ZFP36
cell communication	3072	25	2.061	0.0166	ALDH2, ASB15, CD38, DHCR24, EGFR, EYA1, FAM126A, HSPB1, IFIT1, KIF1B, LGI1, LGR5, MFAP4, MKNK2, MSTN, MYLK2, NUP160, OXCT1, PRKAG3, PYGO1, SLC1A3, TP63, UNC13B, UNC13C, ZFP36
neuron part	387	6	2.722	0.01809	CADM2, KIF1B, MYLK2, SLC1A3, TP63, UNC13C
cofactor binding	184	4	3.023	0.0181	ALDH6A1, DHCR24, DMGDH, GPT2
response to radiation	184	4	3.02	0.01815	EGFR, EYA1, SLC1A3, TP63
negative regulation of apoptotic process	282	5	2.821	0.01855	CD38, EGFR, EYA1, HSPB1, TSC22D1
negative regulation of programmed cell death	287	5	2.775	0.01984	CD38, EGFR, EYA1, HSPB1, TSC22D1
response to light stimulus	106	3	3.214	0.02066	EGFR, SLC1A3, TP63
negative regulation of cell death	300	5	2.659	0.02345	CD38, EGFR, EYA1, HSPB1, TSC22D1
blood circulation	203	4	2.777	0.02484	CD38, EPB41, MYLK2, SMTNL1
circulatory system process	204	4	2.765	0.02523	CD38, EPB41, MYLK2, SMTNL1
muscle tissue development	214	4	2.649	0.02932	EYA1, MSTN, MYLK2, TP63
protein serine/threonine kinase activity	321	5	2.487	0.03006	EGFR, MKNK2, MYLK2, NEK10, PRKAG3
regulation of apoptotic process	688	8	2.224	0.03118	CD38, DHCR24, EGFR, EYA1, HSPB1, TP63, TSC22D1, UNC13B
protein kinase activity	440	6	2.356	0.03149	EGFR, MKNK2, MYLK2, NEK10, POLR2J, PRKAG3
neuron projection	328	5	2.442	0.0323	CADM2, KIF1B, MYLK2, SLC1A3, TP63
extracellular region part	573	7	2.236	0.03425	EGFR, IL17D, IL32, LGI1, MFAP4, MSTN, SLC1A3

response to alcohol	131	3	2.73	0.03516	CD38, MSTN, OXCT1
coenzyme binding	134	3	2.682	0.03709	ALDH6A1, DHCR24, DMGDH
cellular amino acid metabolic process	341	5	2.33	0.03754	AASS, ALDH6A1, DMGDH, GPT2, SLC1A3
negative regulation of hydrolase activity	135	3	2.664	0.03786	DHCR24, IFIT1, SH3RF2
positive regulation of cell proliferation	348	5	2.279	0.04038	CD38, EGFR, EYA1, TP63, TSC22D1
carboxylic acid metabolic process	599	7	2.095	0.04228	AASS, ADHFE1, ALDH6A1, DMGDH, GPT2, PRKAG3, SLC1A3
regulation of multicellular organismal process	1077	11	2.15	0.04274	CD38, EYA1, HSPB1, LGI1, MSTN, MYLK2, SLC1A3, SMTNL1, TP63, UNC13B, ZFP36
epidermis development	143	3	2.539	0.04358	DHCR24, EGFR, TP63
oxidoreductase activity	481	6	2.116	0.04533	AASS, ADHFE1, ALDH2, ALDH6A1, DHCR24, DMGDH
regulation of translation	149	3	2.451	0.04813	HSPB1, MKNK2, PAIP2B
epithelial cell proliferation	150	3	2.437	0.04891	EGFR, EYA1, TP63

*1: Number of genes that are linked to each GO term and GO terms located at the hierarchy under the GO among all of the genes expressed in human skeletal muscle.

*2: Number of genes that are linked to each GO term and GO terms located at the hierarchy under the GO among the 79 significantly upregulated genes after the HIIT intervention.

*3: A value indicating by how much the number of changed genes deviates from the expected value based on the ratio of changed genes to total genes expressed in human skeletal muscle.

*4: The significance probability against the null hypothesis that there is no difference between the ratios of genes with specific GO terms to those without specific GO terms between the changed gene set and the total genes expressed in human skeletal muscle.

Supplemental Table S3. Significantly enriched pathways associated with the HIIT-induced genes

Pathway Name	Total genes*1	Changed genes*2	Z score	*P* value	Gene
Glucose metabolism	59	5	10	4.04E-06	FBP2, GOT1, MDH1, PGK1, PKM
Gluconeogenesis	30	4	11.4	7.86E-06	FBP2, GOT1, MDH1, PGK1
Focal adhesion	197	5	4.9	0.00097	COL4A1, COL4A2, KDR, LAMB1, MYLK4
Arginine and proline metabolism	54	3	6.1	0.00133	CARNS1, CKMT2, GOT1
PI3K-Akt signaling pathway	325	6	4.2	0.00146	COL4A1, COL4A2, FGF6, KDR, LAMB1, SGK1
Insulin signaling pathway	130	4	4.9	0.00166	FBP2, PPARGC1A, PPP1R3C, SREBF1
Glycolysis / Gluconeogenesis	61	3	5.7	0.00186	FBP2, PGK1, PKM
Metabolism of carbohydrates	229	5	4.4	0.00186	FBP2, GOT1, MDH1, PGK1, PKM
Small cell lung cancer	83	3	4.7	0.00432	COL4A1, COL4A2, LAMB1
ECM-receptor interaction	84	3	4.7	0.00446	COL4A1, COL4A2, LAMB1
Integrin cell surface interactions	84	3	4.7	0.00446	COL4A1, COL4A2, LAMB1
Developmental Biology	413	6	3.5	0.00478	COL4A1, COL4A2, LAMB1, NRP1, PPARGC1A, SREBF1
Extracellular matrix organization	92	3	4.4	0.0057	COL4A1, COL4A2, TPSAB1
mTOR signaling pathway	97	3	4.3	0.00658	PPARGC1A, SGK1, SREBF1
Amoebiasis	107	3	4	0.00855	COL4A1, COL4A2, LAMB1
PPARA Activates Gene Expression	110	3	3.9	0.00921	G0S2, PPARGC1A, SREBF1
Regulation of Lipid Metabolism by Peroxisome proliferator-activated receptor alpha (PPARα)	113	3	3.9	0.00989	G0S2, PPARGC1A, SREBF1
Metabolism	1359	11	2.5	0.01384	CA14, CKMT2, FBP2, G0S2, GOT1, MDH1, PGK1, PKM, PPARGC1A, SLC25A15, SREBF1
Fatty acid, triacylglycerol, and ketone body metabolism	171	3	2.9	0.02896	G0S2, PPARGC1A, SREBF1
Signaling by PDGF	178	3	2.8	0.03203	COL4A1, COL4A2, FGF6
Axon guidance	320	4	2.5	0.03491	COL4A1, COL4A2, LAMB1, NRP1
Pathways in cancer	320	4	2.5	0.03491	COL4A1, COL4A2, FGF6, LAMB1
Metabolism of amino acids and derivatives	194	3	2.6	0.03968	CKMT2, GOT1, SLC25A15

*1: Number of genes that are linked to each pathway among all of the genes covered by the Human Gene 2.0 ST Array.

*2: Number of genes that are linked to each pathway among the 79 significantly up-regulated genes after the HIIT intervention.

Supplemetal Table S4. Significantly enriched pathways associated with the genes downregulated by HIIT

Pathway name	Total genes*1	Changed genes*2	Z score	P-value	Gene
Valine, leucine and isoleucine degradation	43	3	6.506	0.001006434	ALDH2, ALDH6A1, OXCT1
Processing of Capped Intron-Containing Pre-mRNA	132	3	3.2	0.02042208	NUP160, POLR2J, SF3A1
Calcium signaling pathway	176	3	2.552	0.042064301	CD38, EGFR, MYLK2
Transmission across Chemical Synapses	182	3	2.48	0.045654063	ALDH2, SLC1A3, UNC13B
Epstein-Barr virus infection	192	3	2.367	0.051967657	CD38, HSPB1, POLR2J
Metabolism of amino acids and derivatives	194	3	2.345	0.053279336	AASS, ALDH6A1, GPT2
Regulation of actin cytoskeleton	207	3	2.21	0.062194954	EGFR, MYLK2, PFN2
MAPK signaling pathway	249	3	1.839	0.095354169	EGFR, HSPB1, MKNK2
Neuronal System	272	3	1.667	0.116064863	ALDH2, SLC1A3, UNC13B
Metabolism	1359	9	1.262	0.176224703	AASS, ADHFE1, ALDH2, ALDH6A1, DHCR24, GPT2, NT5C2, NUP160, OXCT1
Gene Expression	973	6	0.852	0.431867578	HIST2H2AC, HSPB1, NUP160, POLR2J, SF3A1, ZFP36
Disease	784	4	0.313	0.772597598	EGFR, HIST2H2AC, NUP160, POLR2J

*1: Number of genes that are linked to each pathway among all of the genes covered by the Human Gene 2.0 ST Array.

*2: Number of genes that are linked to each pathway among the 73 significantly downregulated genes after the HIIT intervention.

Supplemetal Table S5. Genes correlated with physiological parameter changes by HIIT among 79 up-regulated genes

Maximal oxygen uptake			Maximal accumulated oxygen deficit			Thigh muscle CSA		
Gene symbol	Correlation coefficient	P value	Gene symbol	Correlation coefficient	P value	Gene symbol	Correlation coefficient	P value
SGK1	-0.629909953	0.037788443	MIR412	0.698065474	0.016901628	CKMT2	0.88214193	0.000326276
SCN4B	-0.606567609	0.047865199	NRP1	0.662103995	0.026454766	GOT1	0.878127013	0.000377085
			FARP1	-0.649366126	0.030602505	RPL13P5	0.821584047	0.001923402
			FLJ31813	0.64111457	0.033521063	FBP2	0.753529777	0.007406767
			DNM1P35	-0.602783292	0.049656664	LOC100131174	0.728033416	0.011078419
						PPARGC1A	0.724849545	0.011615077
						ATP5G3	0.706017337	0.015181768
						PKM	0.70430285	0.015541426
						CA14	0.69161471	0.018396029
						KDR	0.68401179	0.020275939
						MDH1	0.659421098	0.027293203
						PPP1R3C	0.642462166	0.033031643
						NRP1	0.625531069	0.039554342

Correlation coefficient was calculated between the rate of change in physiological parameters and that in gene expression levels.

Supplemetal Table S6. Genes correlated with physiological parameter changes by HIIT among 73 down-regulated genes

Maximal oxygen uptake			Maximal accumulated oxygen deficit			Thigh muscle CSA		
Gene symbol	Correlation coefficient	P value	Gene symbol	Correlation coefficient	P value	Gene symbol	Correlation coefficient	P value
EPB41	-0.65857865	0.027560299	ZNF844	-0.88075287	0.000343226	MSTN	-0.809020477	0.002562553
MIR4681	0.631013627	0.03735217	UNC13C	-0.713654147	0.01365213	PDLIM3	-0.619885123	0.041914742
CD38	-0.621432993	0.04125817	SLC1A3	0.669812286	0.024147233	IFIT1	-0.603030045	0.049538472
IL32	-0.604286972	0.048939416	MKNK2	-0.642461503	0.033031883			
			EGFR	-0.618505143	0.04250617			

Correlation coefficient was calculated between the rate of change in physiological parameters and that in gene expression levels.

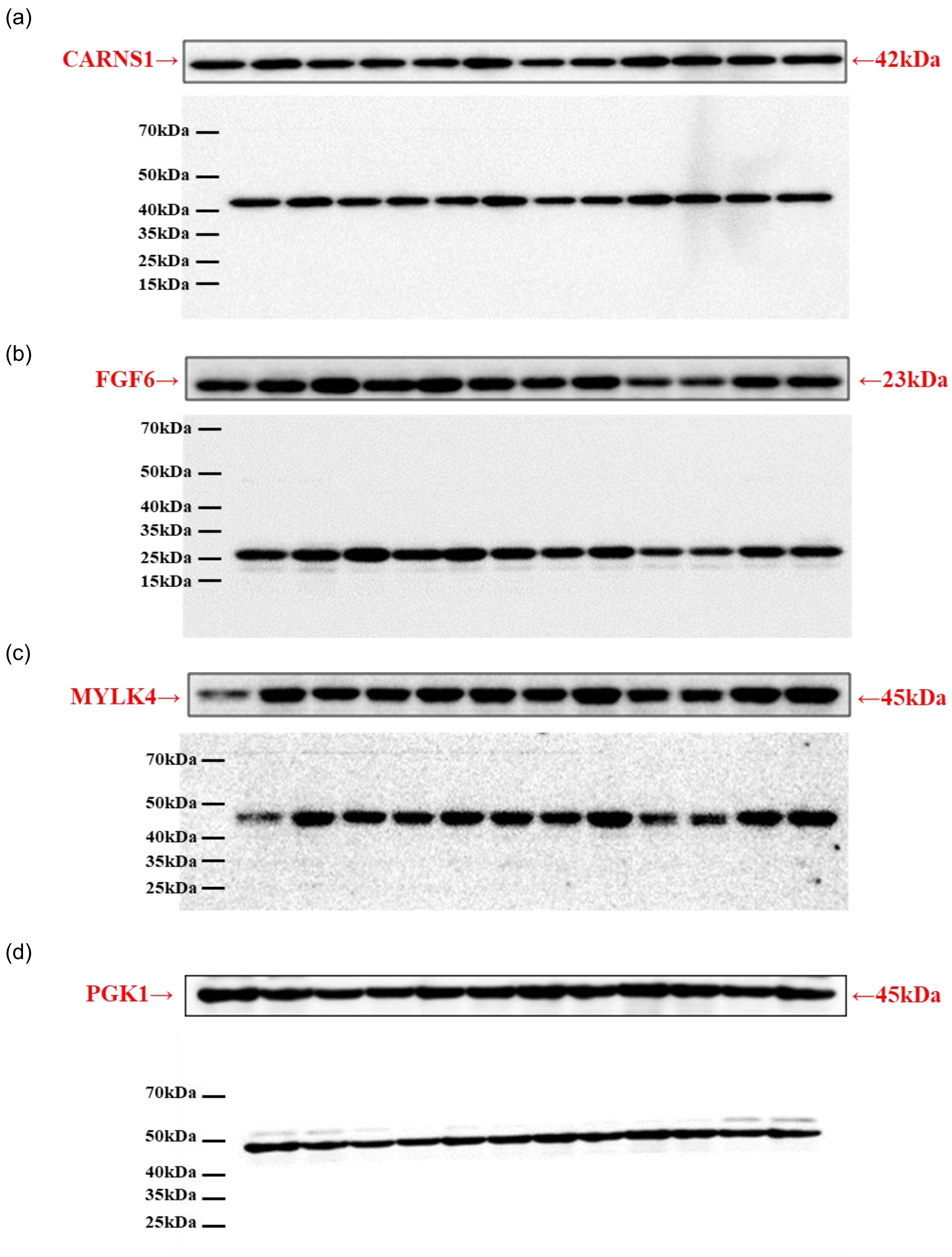

Supplemental Figure S1. Full image of the gels from Figure 3. CARNS1 (a), FGF6 (b), MYLK4 (c), PGK1 (d), PPP1R3C (e), SGK1 (f), PPARGC1A (g), and β-actin (h)

(e)

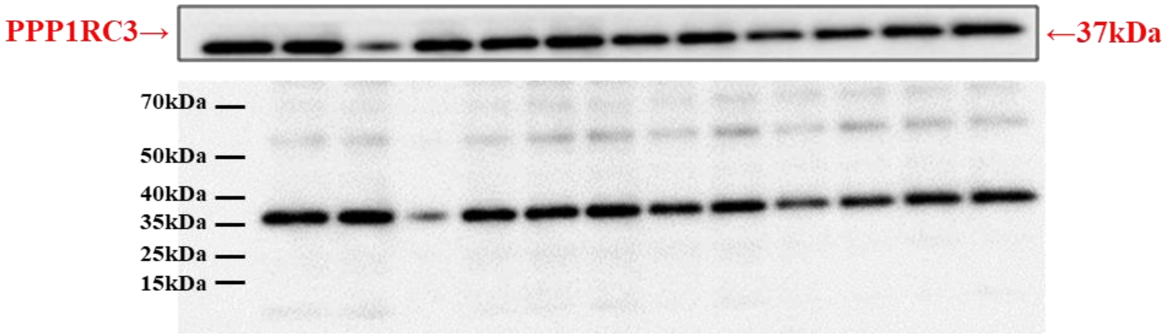

(f)

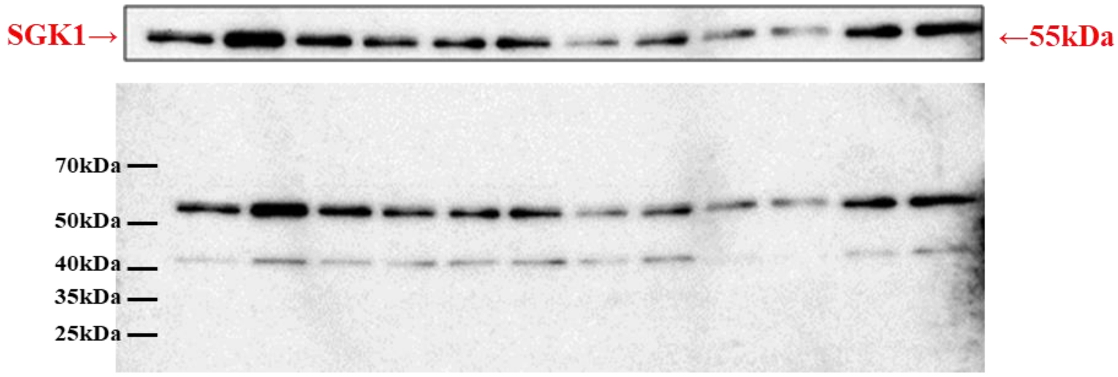

(g)

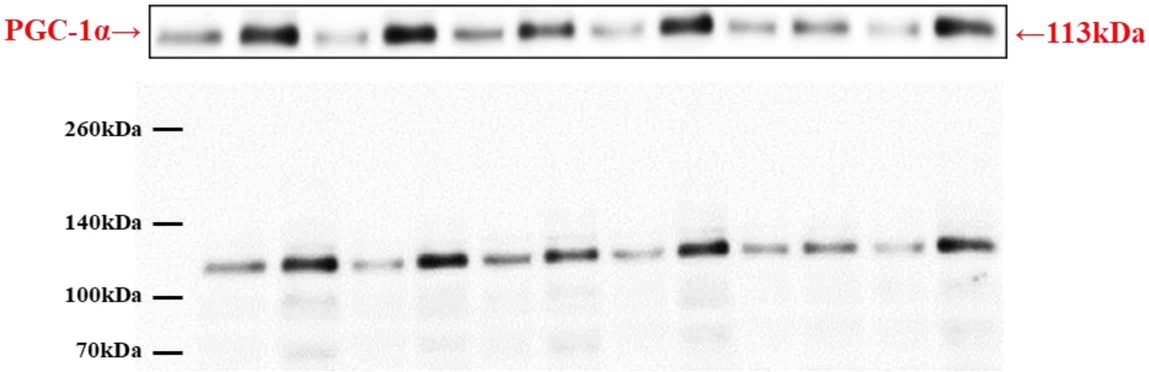

(h)

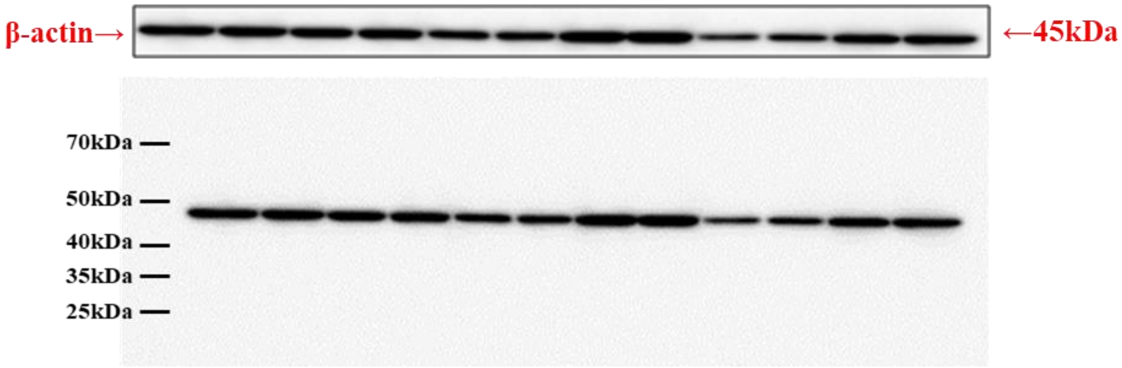

Supplemental Figure S1. Full image of the gels from Figure 3. CARNS1 (a), FGF6 (b), MYLK4 (c), PGK1 (d), PPP1R3C (e), SGK1 (f), PPARGC1A (g), and β-actin (h)

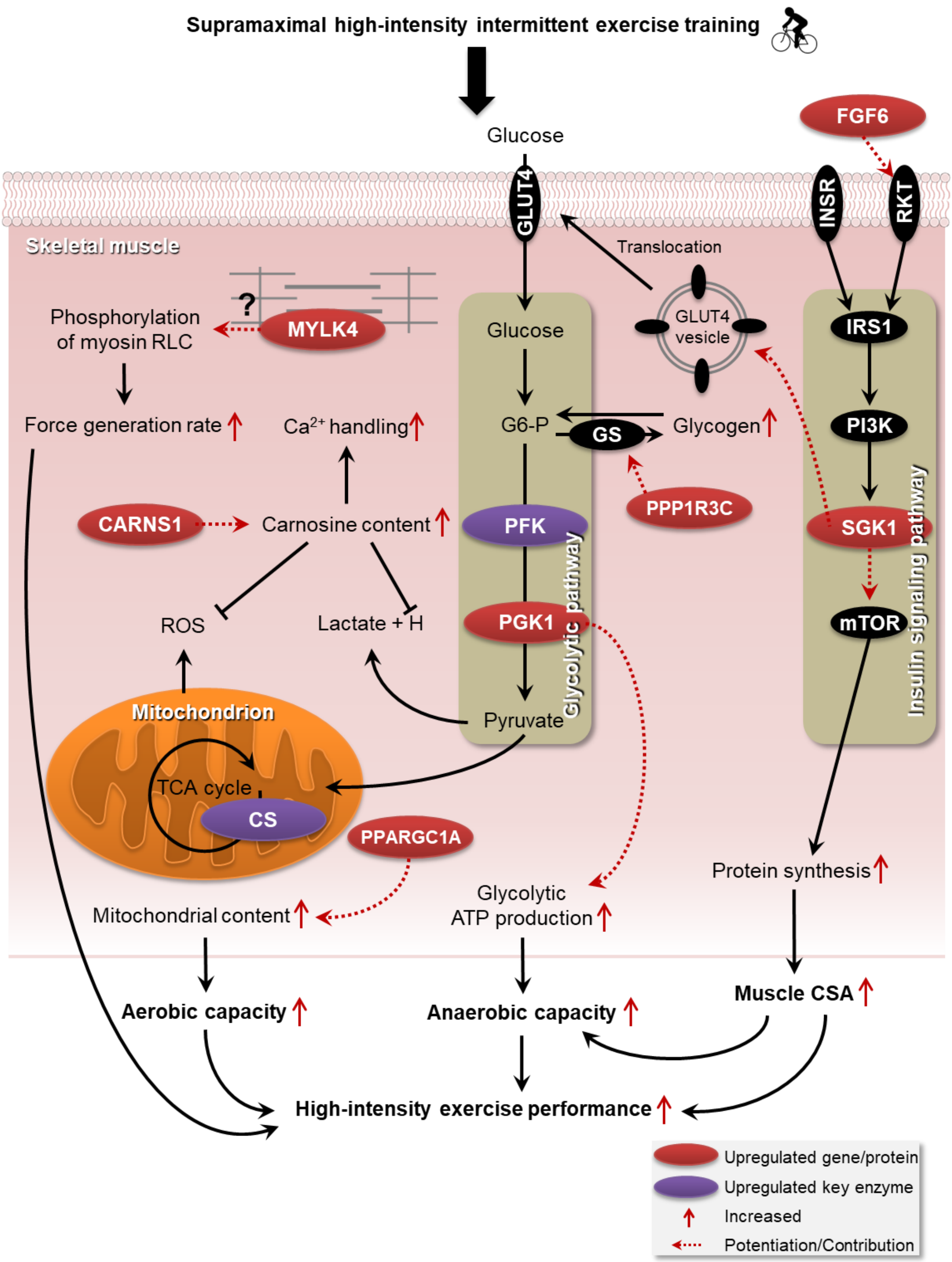

Supplemental Figure S2. Potential roles of upregulated genes/proteins on skeletal muscle adaptations to supramaximal HIIT
